# Does diet breadth affect the complexity of the phytophagous insect microbiota? The case study of Chrysomelidae

**DOI:** 10.1111/1462-2920.15847

**Published:** 2021-11-30

**Authors:** Matteo Brunetti, Giulia Magoga, Fabrizia Gionechetti, Alessio De Biase, Matteo Montagna

**Affiliations:** ^1^ Department of Agricultural and Environmental Sciences University of Milan, Via Celoria 2 Milan 20133 Italy; ^2^ Department of Life Sciences University of Trieste Trieste 34127 Italy; ^3^ Department of Biology and Biotechnology "Charles Darwin" Sapienza University of Rome, Viale dell'Università 32 Rome 00185 Italy; ^4^ BAT Center ‐ Interuniversity Center for Studies on Bioinspired Agro‐Environmental Technology University of Napoli "Federico II" Portici Italy

## Abstract

Chrysomelidae is a family of phytophagous insects with a highly variable degree of trophic specialization. The aim of this study is to test whether species feeding on different plants (generalists) harbour more complex microbiotas than those feeding on a few or a single plant species (specialists). The microbiota of representative leaf beetle species was characterized with a metabarcoding approach targeting V1–V2 and V4 regions of the bacterial 16S rRNA. Almost all the analysed species harbour at least one reproductive manipulator bacteria (e.g., *Wolbachia*, *Rickettsia*). Two putative primary symbionts, previously isolated only from a single species (*Bromius obscurus*), have been detected in two species of the same subfamily, suggesting a widespread symbiosis in Eumolpinae. Surprisingly, the well‐known aphid symbiont *Buchnera* is well represented in the microbiota of *Orsodacne humeralis*. Moreover, in this study, using Hill numbers to dissect the components of the microbiota diversity (abundant and rare bacteria), it has been demonstrated that generalist insect species harbour a more diversified microbiota than specialists. The higher microbiota diversity associated with a wider host‐plant spectrum could be seen as an adaptive trait, conferring new metabolic potential useful to expand the diet breath, or as a result of environmental stochastic acquisition conveyed by diet.

## Introduction

Insects are colonized by a variety of microorganisms, prevalently living as commensals, but which in many cases can confer either beneficial or detrimental effects to their host (e.g., Douglas, 2009; Kikuchi *et al*., 2012; Engel and Moran, 2013; Clay, 2014; Douglas, 2015; Hurst and Frost, 2015; Wang *et al*., 2020). Since symbiont‐mediated traits highly influence the host nutrition, in herbivorous insects this influence is often crucial in the interaction with the host plant (Hansen and Moran, 2014; Giron *et al*., 2017; Mason *et al*., 2019; Frago *et al*., 2020; Mason, 2020). Most of the microorganisms that can be found within the insect body colonize the gut lumen, but they can be also hosted in specialized organs often connected to female genitalia, especially when the vertical transmission of the symbiont is required (Stammer, 1935, 1936; Mann and Crowson, 1983; Becker, 1994). The most specialized bacterial symbionts live inside the insect cells are vertically transmitted, and show drastic genome reduction (e.g., *Blattabacterium*, *Buchnera*), usually maintaining only the metabolic pathways involved in providing functional traits to the host (Boscaro *et al*., 2017; Latorre and Manzano‐Marín, 2017; Ankrah *et al*., 2018). Other bacteria are able to colonize insect cells, such as the reproductive manipulators belonging to the so‐called male‐killing group (e.g., bacteria of the genera *Wolbachia* and *Rickettsia*) that can manipulate the host reproduction to maintain their infection across generations and spread within the population (Harris *et al*., 2010; Correa and Ballard, 2016; Larracuente and Meller, 2016).

Most studies investigating the relationship between bacterial symbionts and the insect host have been conducted on model species, mainly focusing on single interactions. More recently the advent of next‐generation sequencing techniques coupled with the 16S rRNA‐based approach for the bacterial taxonomy has greatly facilitated the characterization of the full microbiota associated with non‐model organisms, allowing to expand the experimental scale (e.g., Montagna *et al*., 2015a; Mohammed *et al*., 2018; Ziganshina *et al*., 2018; Kolasa *et al*., 2019). This innovation opened the possibility to characterize the microbiota associated with several wild species and so to investigate the correlations between the composition of microbial communities and several ecological or physiological traits of the insect host, such as the breadth of the insect diet (Colman *et al*., 2012; Yun *et al*., 2014). Indeed, insects feeding on several plant species may be expected to harbour more complex microbial communities. The gut microbiota composition can be influenced by the diet, directly since food may inoculate bacteria able to colonize the insect gut or indirectly by promoting the growth of specific bacteria (Pérez‐Cobas *et al*., 2015; Montagna *et al*., 2015b; Chouaia *et al*., 2019; Muturi *et al*., 2019). So, insects with a wider food source spectrum are expected to be colonized by a higher diversity of microbial taxa. Anyway, the higher diversity in the microbiota of generalist species could also be due to the wider metabolic potential, conferred by the presence of a more variegated microbial community, which makes those insects able to exploit several different food sources. The covariation of microbiota diversity and breadth of the animal diet has been investigated also in non‐insect taxa, usually achieving inconclusive results that do not support the hypothesis of a higher diversity in the microbiota of generalist species (e.g., Kartzinel *et al*., 2019; Chen *et al*., 2021).

Leaf beetles (Coleoptera: Chrysomelidae), including ~40 000 species worldwide, constitute one of the most diverse insect groups in the world (Leschen and Beutel, 2014). This Coleoptera family includes almost only phytophagous species, feeding on leaves or other plant organs at least at the adult stage. The degree of trophic specialization is highly variable, since some leaf beetle species can exploit only one or few specific plant species as a food source, while others can feed on hundreds of plant species belonging to several different families. This makes Chrysomelidae a perfect model to investigate the relationship between the level of microbiota complexity and the breadth of the host plant spectrum. Furthermore, leaf beetles are of great interest for the presence of vertically transmitted symbionts (i.e., in Donacinae, in Cassidinae and in Eumolpinae), which are harboured in specialized host organs associated with gut and genitalia (Stammer, 1935, 1936; Tayade *et al*., 1975; Mann and Crowson, 1983; Becker, 1994). Bacteria of the genus ‘*Candidatus* Macropleicola’ (Enterobacteriaceae) are hosted in specialized organs at the midgut–hindgut junction of Donacinae. These bacteria show a tight co‐speciation with the insect host and are involved in supporting its nutrition providing essential nutrients during the larval stage (essential amino acids, riboflavin) and digestive enzymes (pectinases) to the adult insects (Kölsch *et al*., 2009; Kölsch and Pedersen, 2010; Kleinschmidt and Kölsch, 2011; Reis *et al*., 2020). Similarly, ‘*Candidatus* Stammera capleta’ (Enterobacteriaceae), hosted in specialized organs associated with the foregut of several Cassidinae species, is involved in pectinase production (Salem *et al*., 2017, 2020). Within Eumolpinae only a single species is known to host symbionts in specialized organs, *Bromius obscurus* (Stammer, 1936). This symbiosis has been less studied, but two different symbionts have been isolated from it (Kölsch and Synefiaridou, 2012). The first one (henceforth *B*. *obscurus* symbiont A) is hosted intracellularly in blind sacs at the foregut–midgut junction and extracellularly in female‐specific genital accessory organs, suggesting the presence of vertical transmission. The second one (henceforth *B*. *obscurus* symbiont B) is hosted in small crypts at the end of the midgut and is phylogenetically related to bacteria species living in the gut lumen, not tightly associated with the host (Kölsch and Synefiaridou, 2012; Fukumori *et al*., 2017).

Previous studies on the bacteria associated to leaf beetles were mainly focused on reproductive manipulators (Clark *et al*., 2001; Keller *et al*., 2004; Kondo *et al*., 2011; Roehrdanz and Wichmann, 2013; Montagna *et al*., 2014; Krawczyk *et al*., 2015; Kolasa *et al*., 2017; Takano *et al*., 2017; Gómez‐Zurita, 2019), single species of economic importance (Muratoglu *et al*., 2011; Chung *et al*., 2013; Ali *et al*., 2019; Ludwick *et al*., 2019; Wang *et al*., 2019; Shukla and Beran, 2020) or few strictly related species (Kelley and Dobler, 2011; Montagna *et al*., 2015a; Blankenchip *et al*., 2018; Wei *et al*., 2020). The present study aims to characterize the microbiota associated with a selection of leaf beetle species, representative of the taxonomic diversity and of the various degrees of trophic specialization. In detail, it aims: (i) to determine the principal bacterial taxa that characterize the microbiota of the selected leaf beetle species, also detecting the presence of important insect symbionts (e.g., *Wolbachia*) and symbionts typically present in specific Chrysomelidae subfamilies (e.g., Donacinae, Cassidinae); (ii) to test the hypothesis that the microbiota of generalist phytophagous species is more complex than the microbiota of more specialist species.

## Results

### Efficiency and taxonomic resolution of the 16S rRNA gene V1–V2 and V4 regions

From the 30 Chrysomelidae species analysed a total of 841 822 (mean per sample = 28 060.7) and 1 711 075 (mean per sample = 57 035.8) raw reads have been obtained from the sequencing of the V1–V2 and V4 regions of the bacterial 16S rRNA respectively. Raw sequences have been deposited on the NCBI SRA database under the project accession number PRJNA729224. After the denoising and filtering steps, the V1–V2 dataset consisted of 1080 amplicon sequence variants (ASVs) (total reads = 449 368; mean per sample = 14 978.9) and the V4 dataset consisted of 1572 ASVs (total reads = 1 047 226; mean per sample = 34 907.5). All the ASVs assigned to mitochondria (<0.01% of the V1–V2 reads, 0.74% of the V4 reads) or chloroplast (45.3% of the V1–V2 reads, 17.8% of the V4 reads) have been excluded from further analyses. Regarding the taxonomic identification of the ASVs, 119 bacterial genera have been identified by both regions while 162 genera are present only in the V4 dataset and 35 genera only in the V1–V2 dataset. Comparing the results of the taxonomic assignment of the two regions, the V4 region results the marker almost always more efficient in detecting bacterial taxa (Supplementary Fig. 1), with few exceptions in which those for the V1–V2 region slightly outperform the others (e.g., the genus *Brevundimonas* and *Aeromonas*). The estimated diversity using the two regions separately (Supplementary Fig. 2) is identical when putting much weight on the most abundant species (*q* = 2), while the V4 region provides slightly higher estimates when increasing the weight of rare species (*q* = 1, *q* = 0).

### Microbiota composition

**Fig. 1:**
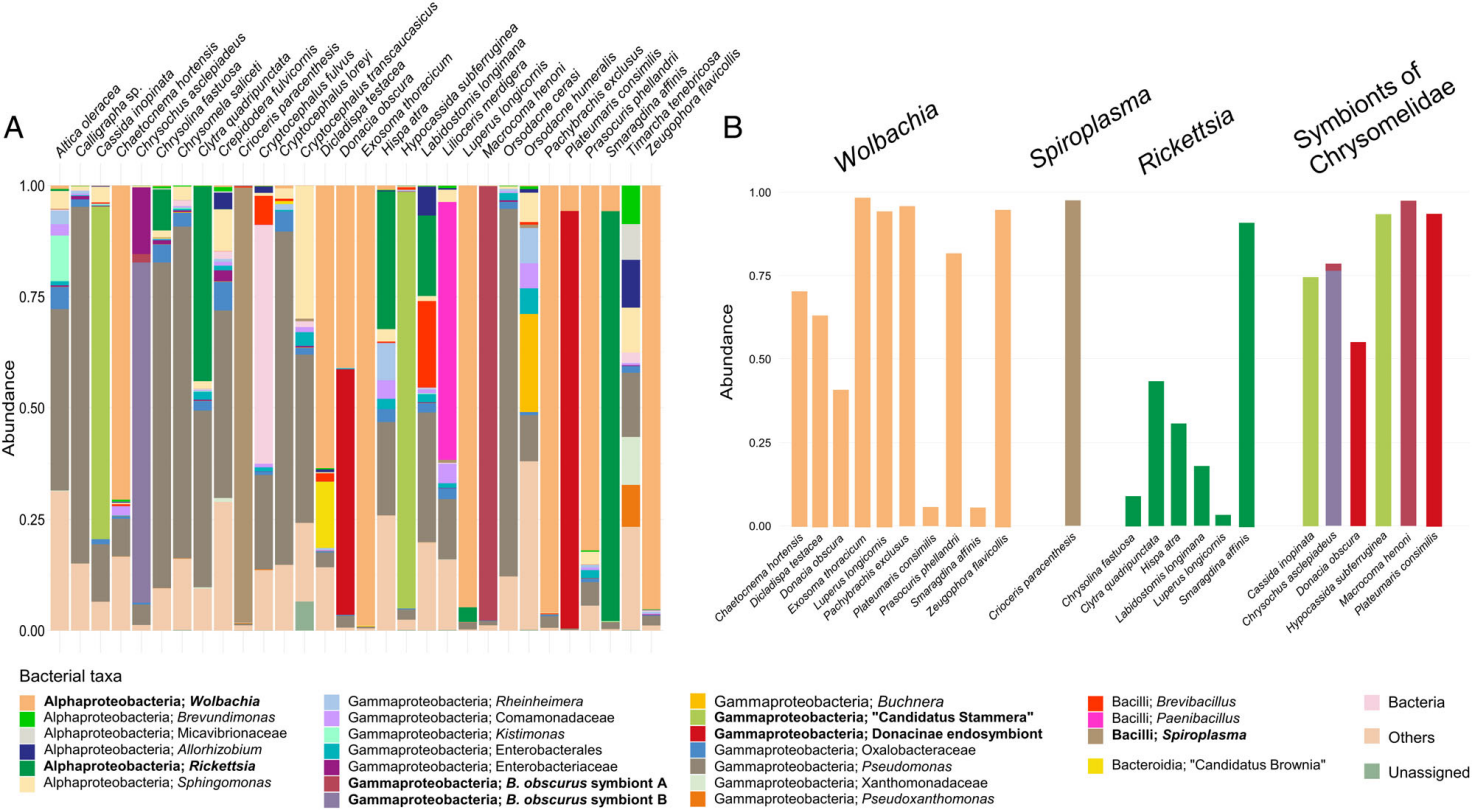
Taxonomic composition of the bacterial microbiota of Chrysomelidae. A. Bar plot representing the composition of the microbiota of each Chrysomelidae species at the genus level (or any higher taxonomic level when the identification at the genus level was not possible). Colours represent different bacterial ranks, as reported in the legend, and the height of each box corresponds to the average relative abundance of each bacterial rank. Only bacterial ranks representing on average at least 5% of the reads in one species are shown, less abundant bacteria are included in the group ‘Others’. B. Relative abundance of reproductive manipulators (*Wolbachia*, *Rickettsia*, *Spiroplasma*) and bacterial symbionts present only in Chrysomelidae. Colours representing different bacterial genera are reported in the legend (in bold). Only insect species with a relative abundance of these bacteria of at least 3% are shown.

**Fig. 2:**
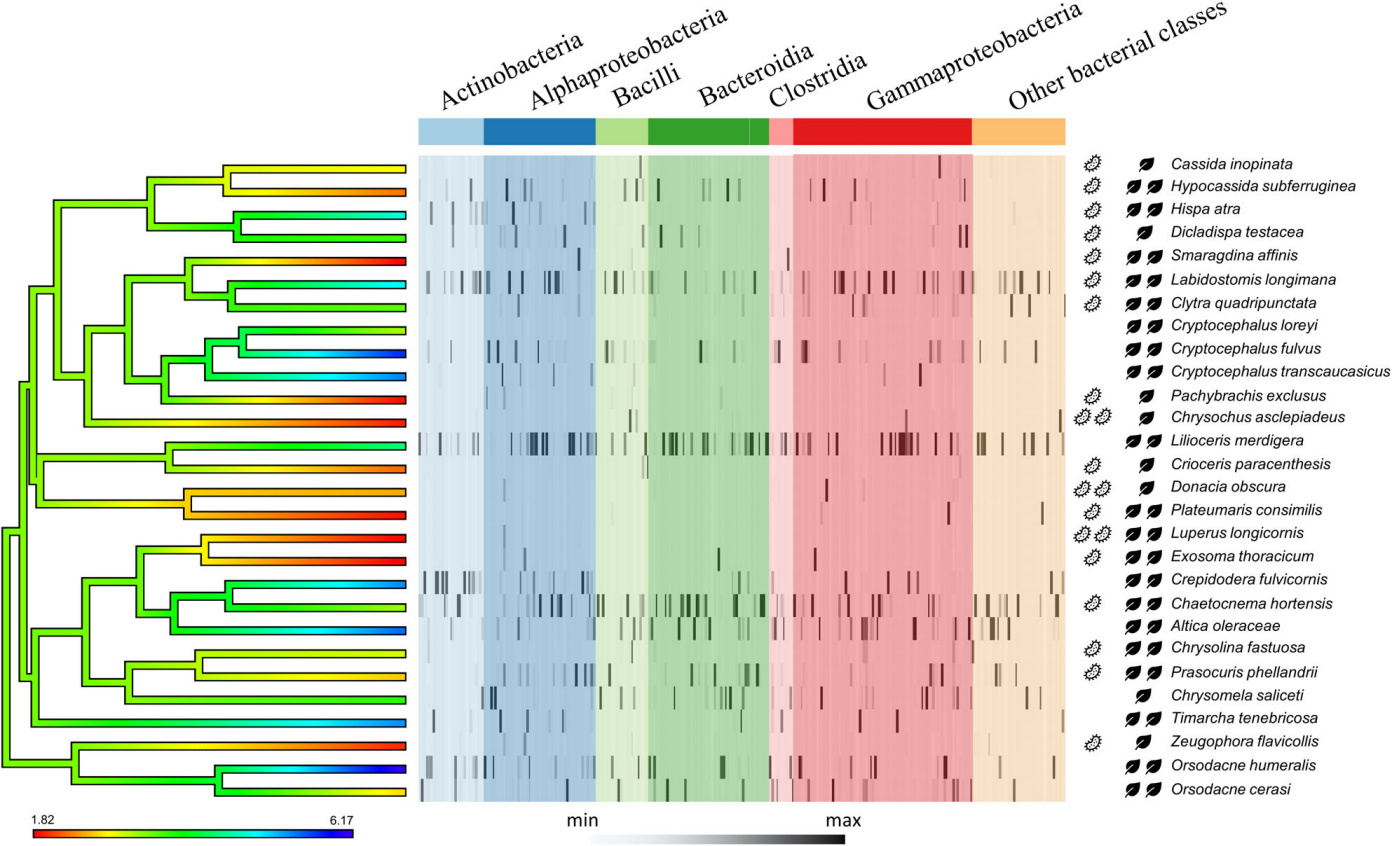
Diversity and composition of the Chrysomelidae microbiota. On the left side the ancestral state reconstruction of the microbiota diversity (Hill numbers, *q* = 1) is plotted as a colour gradient on the ML phylogenetic tree of the selected Chrysomelidae species. The heatmap represents the relative abundance of each genus with colours corresponding to the bacterial class (only classes with at least five different bacterial genera are reported, other classes are included in the category ‘Other bacterial classes’). On the right side, together with the insect species names, the presence of one or more reproductive manipulators or Chrysomelidae specific symbionts (one or two bacterium icons) and the trophic classification of the insect (specialists with one leaf icon; generalists with two leaf icons) are reported.

The most represented bacterial classes associated to the analysed Chrysomelidae species (Figs 1A and 2) are Alphaproteobacteria (~39%), Gammaproteobacteria (~45%) and Bacilli (~14%). Several genera belonging to Bacteroidia are also present in the microbiota of the selected species (Fig. 2) but this class constitutes only ~1% of the total dataset. Within Alphaproteobacteria the most abundant genera recorded are *Wolbachia*, *Rickettsia* and *Sphingomonas*. *Wolbachia* is the most represented genus in the dataset (~30% of the total reads) and sequences belonging to this genus have been found in all the species except *Chrysomela saliceti* and *Timarcha tenebricosa* (Fig. 1B, Supplementary Table 1). In most cases *Wolbachia* sequences represent a low percentage of the sample reads (<1%), while in eight species it comprises the most abundant ASVs. *Rickettsia* is another well‐represented genus (Fig. 1B, Supplementary Table 1). Reads assigned to *Rickettsia* have been found in nine species and represents a high percentage of the reads from *Hispa atra* (~31%) and from all the sampled species belonging to Clytrini tribe (*Labidostomis longimana* ~18%, *Clytra quadripunctata* ~44%, *Smaragdina affinis* ~92%). *Sphingomonas* is also quite common in the dataset (Fig. 1B). It is present in all the sampled species, with the only exception of *Plateumaris consimilis*, and it reaches the highest densities in *Timarcha tenebricosa* (~10%) and *Cryptocephalus transcaucasicus* (~30%). Within Gammaproteobacteria the most abundant genus is *Pseudomonas* (Fig. 1A). *Pseudomonas* is the second most abundant genus in the dataset (~12% of the total reads) and it is the only bacterial genus that is present in all the sampled species, with relative abundances that varies from 0.3% to 82%. In total 141 ASVs (corresponding to 15 97%‐similarity OTUs) are assigned to *Pseudomonas*, 69 of them are present only in one species so that 21 species have at least one unique ASV assigned to this genus. Another surprisingly well‐represented bacterial genus belonging to Gammaproteobacteria is *Buchnera* (Fig. 1A). It has been found in 21 species, mostly at low abundance but representing a quite high proportion of the reads obtained from *Orsodacne humeralis* (~22%). The most abundant genus belonging to Bacilli is *Spiroplasma*, followed by *Paenibacillus* and *Brevibacillus* (Fig. 1A). *Spiroplasma* is the dominant genus in *Crioceris paracentesis* (~98%) and it has been found also in 18 other species, but with low densities (Fig. 1B). While in the microbiota of *Lilioceris merdigera* the dominant genus is *Paenibacillus* (~58%), that is also present in few other species but with low abundances.

The results of the NCBI blast search (Supplementary Table 2) and phylogenetic tree inference (Supplementary Fig. 3) shed further light on the bacterial taxa that characterize the microbiota of Chrysomelidae. Two ASVs, which have been found only in *Cryptocephalus fulvus* (53.6%), have been assigned by the naïve Bayes classifier (confidence >0.95) only to the domain level and the top hits of the blast search (query coverage 100%, identity >80%) correspond to uncultured bacteria isolated from acidic biofilm in caves (DQ499258). A huge number of sequences obtained from Donacinae (*Donacia obscura* 55.3%, *Plateumaris consimilis* 93.8%) belong, with high confidence (CP046230; query coverage 100%, identity 99.7%), to the vertically transmitted endosymbiont widespread in this subfamily (Kölsch *et al*., 2009). In both the maximum likelihood (ML) trees those sequences cluster with sequences obtained from the bacterial symbiont of Donacinae with quite high confidence (bootstrap values >70). Similarly, most of the sequences obtained from Cassidinae (*Cassida inopinata* 74.7%, *Hypocassida subferruginea* 93.1%) resulted to belong to ‘*Candidatus* Stammera capleta’ (CP024013; query coverage 100%, identity 98.8%) and cluster with sequences of this species in the ML trees with high confidence (bootstrap value =100). Three ASVs from the V1–V2 region assigned to Enterobacterales are present only in *Macrocoma henoni* (23.9% of the reads). Blast search top hits (query coverage 100%, identity >78%) correspond to endosymbionts of weevils (AP018159, KX067892) while in the ML tree they cluster with a sequence from the *B*. *obscurus* symbiont A (LC273302) with high confidence (bootstrap value =99). Also, two ASVs from the V4 region from *M*. *henoni* (that represent almost all the reads previously assigned to *Buchnera* in this species) clustered together with sequences of the *B*. *obscurus* symbiont A (bootstrap value =88; Supplementary Fig. 3); the blast search confirms this taxonomic annotation (query coverage 100%, identity 93.9% and 93.5% with LC273302 and JQ805030 respectively). Six ASVs are present only in *Chrysochus asclepiadeus* and represent 93.3% of the reads from this species, one of them can be assigned to the *B*. *obscurus* symbiont A (LC273302, JQ805030; query coverage 99%, identity >87%, bootstrap value =97). Among the remaining ASVs blast search top hits assigned two ASVs to *Lelliottia amnigena* (LR134135; query coverage 100%, identity >98%) and the other three ASVs to *Klebsiella* sp. (MN860163, LR134475, MT279983, MT255043; query coverage 100%, identity >98%). In the ML trees (Supplementary Fig. 3) those sequences are part of a clade that includes *Klebsiella* and *Lelliottia*, but also other bacterial genera including symbionts of Hemiptera (JQ322760, HM156667, AB650515, AY620432) and the *B*. *obscurus* symbiont B (JQ805033).

### Microbiota diversity

**Fig. 3:**
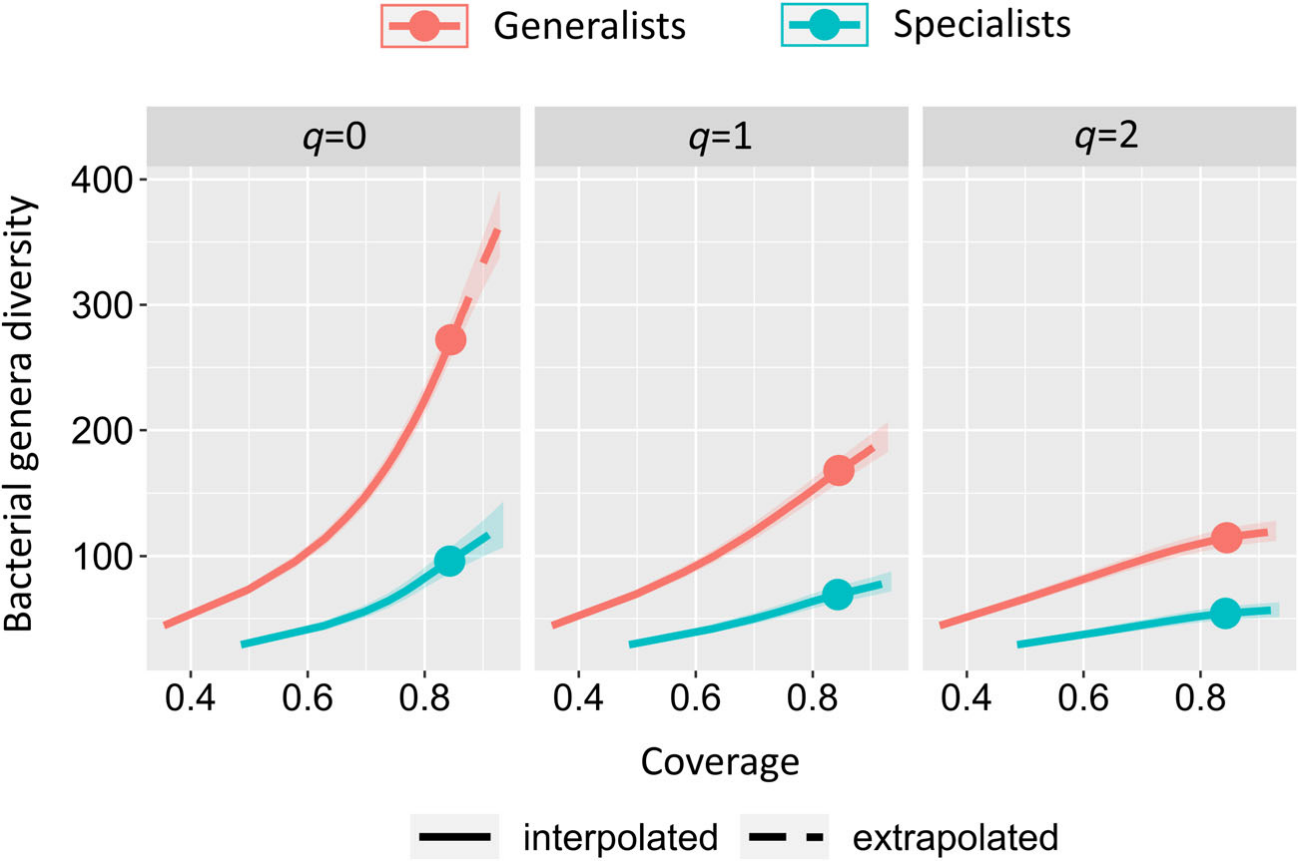
Microbiota diversity of specialist and generalist Chrysomelidae defined using the plant taxonomic level of family (specialists feed on plants all belonging to the same family, generalists feed on plants belonging to different families). Coverage based rarefaction/extrapolation curves of the Hill numbers estimated for three values of the order parameter (*q* = 0, *q* = 1, *q* = 2). The *x*‐axis represents the coverage (that estimates the completeness of the sampling) and the *y*‐axis represents the Hill number estimates, 95% confidence interval is also reported. As reported in the legend, colours correspond to the trophic category (specialist or generalist) and line type to the methodological approach (interpolation or extrapolation).

Diversity estimates for the group of generalist species, identified as those feeding on several plant families, are always higher than estimates for specialist species (Fig. 3; Supplementary Fig. 2). Similar results have been obtained also defining the two trophic groups (i.e., generalists, specialists) by working at the level of plant genera (Supplementary Fig. 4). As an example, with *q* = 2 (i.e., counting mainly the dominant taxa) the diversity estimated for generalist species is almost twice the diversity estimated for specialist species (coverage >0.3). Also, the diversity partitioning analysis in the framework of Hill numbers confirms this pattern (Table 2). The α‐diversity component (average diversity of single species microbiotas) is always higher in generalist species, regardless of the value of the order parameter (*q*). Also, the γ‐diversity (diversity of the microbiota of all the species together) is higher in generalist species, except in the case of *q* = 0 (i.e., counting mainly the rare species). When using an intermediate weight (*q* = 1; counting mainly the common species) the gamma diversity estimated for the entire dataset (5.5) has an intermediate value between specialist species (4.9) and generalist species (6.4), as expected, while using other values for the order parameter there are no clear differences. The β‐diversity estimates (γ‐diversity/α‐diversity; corresponding to differences between samples) are similar in generalists and specialists, except for *q* = 0.

The ancestral state reconstructions of the microbiota diversity estimates along the Chrysomelidae phylogenetic tree show no clear pattern (Fig. 2), in fact, no phylogenetic signal has been recorded. Some phylogenetic clades share low levels of microbial diversity (e.g., Donacinae, Cassidinae) but it is probably related to the presence of primary symbionts at high abundances (Fig. 2). In fact, most of the species hosting primary symbionts (e.g., ‘*Candidatus* Stammera capleta’) or reproductive manipulators (e.g., *Wolbachia*) have low microbial diversity estimates (especially for *q* < 2). In any case, there are still several species hosting reproductive manipulators that have highly diverse microbiota (e.g., *Labidostomis longimana*, *Chaetocnema hortensis*).

## Discussion

### Efficiency and taxonomic resolution of the 16S rRNA gene V1–V2 and V4 regions

The V4 region of the 16S rRNA, one of the two markers used in this study to characterize the microbiota associated to Chrysomelidae, allowed to obtain a higher number of ASVs and seems less prone to chloroplast contamination in respect to the second marker adopted, the V1–V2 region. Moreover, several bacterial genera were identified only through the examination of V4 region reads (Supplementary Fig. 1) and the diversity analyses suggest that this region better recovers rare taxa (Supplementary Fig. 2). These results are in accordance with what was found in previous studies supporting the use of the V4 region of the 16S rRNA gene in metabarcoding studies on bacteria (Zhang *et al*., 2018; Chen *et al*., 2019). Nevertheless, the V4 region missed the amplification of some bacterial taxa (e.g., *Brevundimonas* and *Aeromonas*) and the values of the diversity indices obtained combining the two regions resulted higher than those obtained from a single region (Supplementary Fig. 2). These results support the use of multiple marker regions to increase the resolution of metabarcoding studies targeting bacterial communities.

### Microbiota composition

Within the microbiota associated with the 30 species of Chrysomelidae analysed in this study, three endosymbiotic bacterial genera belonging to the so‐called male‐killing group (Engelstädter and Hurst, 2009) have been identified: *Wolbachia* (in 28 species), *Spiroplasma* (in 17 species) and *Rickettsia* (in nine species) (Supplementary Table 1). For the majority of the analysed Chrysomelidae, the association with these bacteria is reported in this study for the first time. The presence of reproductive manipulators, such as *Wolbachia*, in Chrysomelidae is well known (Montagna *et al*., 2014; Kajtoch and Kotásková, 2018; Gómez‐Zurita, 2019). These endosymbiotic bacteria are usually abundant in infected species, tending to dominate the community, as observed in this study for four Chrysomelidae species, where *Wolbachia* represent 94%–99% of the reads. Endosymbionts can also represent only a minimum fraction of the bacterial community, e.g., *Wolbachia* represent less than 0.05% of the reads in nine species analysed in this study. The latter cases could be signs of horizontal acquisition that did not lead to an infection (Rasgon *et al*., 2006; Pietri *et al*., 2016; Chrostek *et al*., 2017; Kolasa *et al*., 2017; Cardoso and Gómez‐Zurita, 2020) rather than real infections able to produce effects on the host even with a low bacterial titre (Richardson *et al*., 2019). Surprisingly, *Orsodacne humeralis* was found to host *Buchnera* (Gammaproteobacteria: Enterobacteraceae) representing the 22.1% of bacterial reads obtained for this species. This bacterium is a well‐known endosymbiont that is strictly associated with aphids (Buchner, 1965; Shigenobu and Wilson, 2011) and to our knowledge infections caused by it in a non‐aphid host have never been reported. For this reason, it is also hard to determine the relationship between *Buchnera* and *O*. *humeralis* microbiota (e.g., acquisition from the environment, commensality, symbiosis). *Pseudomonas* is the second most abundant bacterial genus found to be associated with the Chrysomelidae species of this study and it is the only one detected in all analysed species. Species of this genus can live under diverse environmental conditions; they are ubiquitous in soil, water and are important pathogens of plants and animals (Moore *et al*., 2006). *Pseudomonas* species are also known to play a functional role in insect symbiosis (e.g., providing digestive enzymes) (Piel *et al*., 2004; Huang *et al*., 2012; Ceja‐Navarro *et al*., 2015; Zhang *et al*., 2020a,b). The high variety of *Pseudomonas* species makes it difficult to distinguish among environmental contamination (presumably from the food source), facultative association and functional symbiosis. Nevertheless, the high prevalence and uniqueness of *Pseudomonas* ASVs in some samples allows to suppose that, at least in these cases, it could represent a symbiont potentially playing a functional role for some Chrysomeldiae species. Three Chrysomelidae subfamilies (Donacinae, Cassidinae, Eumolpinae) are known to host specific bacterial symbionts in specialized organs associated with the gut. Symbionts of Donacinae and Cassidinae have been intensively studied in the last years and are known to support host nutrition supplying digestive enzymes and/or providing essential nutrients lacking in the insect diet (Kleinschmidt and Kölsch, 2011; Salem *et al*., 2017, 2020; Reis *et al*., 2020). While for Eumoplinae those kinds of symbiosis have been less studied and are known only in *B*. *obscurus*. In both the species of Donacinae included in this study the microbiota is dominated by an endosymbiont already known to be widespread within the species of the subfamily (Fig. 1B, Supplementary Table 1). Specifically, in *Plateumaris consimilis* ~94% of the sequences are assigned to the symbiont isolated from that same species in Reis *et al*. (2020), while in *Donacia obscura* ~55% of the sequences have been assigned to the symbiont isolated in the same study from *Donacia cinerea* and *Donacia marginata*, since no reference sequences are available for *Donacia obscura* symbiont. Most of the reads obtained from both the species of Cassidinae included in this study, *Cassida inopinata* (74.7%) and *Hypocassida subferruginea* (93.1%), have been assigned to symbiont of Cassidinae ‘*Candidatus* Stammera capleta’ (Stammer, 1936; Salem *et al*., 2017) (Fig. 1B, Supplementary Table 1). This result is the first report of ‘*Candidatus* Stammera capleta’ in these two Cassidinae species. The two analysed species of Eumolpinae (*M*. *henoni* and *Chrysochus asclepiadeus*) host bacterial taxa previously reported only from *B*. *obscurus* (*B*. *obscurus* symbiont A and B). The microbiota of *M*. *henoni* is dominated by the *B*. *obscurus* symbiont A. Since in *B*. *obscurus* this intracellular bacterium is present in specialized gut organs and in the female genitalia, it is possible to hypothesize a similar localization and a vertical transmission mechanism also in *M*. *henoni* but further studies are needed to investigate this symbiotic relationship and confirm this hypothesis. The symbiont A of *B*. *obscurus* is also present in *Chrysochus asclepiadeus*, but with a low abundance (~2%). The microbiota of *Chrysochus asclepiadeus* is dominated by a group of closely related bacterial taxa including the *B*. *obscurus* symbiont B together with the two bacterial genera *Lelliottia* and *Klebsiella*. *Lelliottia* spp. are usually isolated from plants, water and clinical samples (Brady *et al*., 2013). This genus has been also found in insect microbiota but usually at low abundances (e.g., Wang *et al*., 2019; Xu *et al*., 2019). *Klebsiella* spp. are often isolated from a variety of environmental sources such as soil, vegetation, water and animals (Brisse *et al*., 2006), but some species are also known to play functional roles in insect symbiosis (e.g., providing enzymes and antibiotics) (Dillon *et al*., 2002; Dantur *et al*., 2015; Miyashita *et al*., 2015). The high prevalence of ASVs closely related to the *B*. *obscurus* symbiont B in *Chrysochus asclepiadeus* suggests the presence of similar symbioses in closely related Eumolpinae species, which should be further investigated.

Interestingly, in the 16S rRNA phylogenetic analyses (Supplementary Fig. 3) ‘*Candidatus* Stammera capleta’, the endosymbiont of Donacinae and the *B*. *obscurus* symbiont A (together with five *M*. *henoni* ASVs and one *Chrysochus asclepiadeus* ASV) clustered in a clade with the most specialized Enterobacteriaceae symbionts (e.g., *Buchnera*, *Blochmannia*, *Nasonia*, *Baumannia*). However, the symbiont B of *B*. *obscurus* is placed in a separate clade with more generalist bacteria (e.g., *Escherichia*, *Enterobacter*, *Serratia*, *Klebsiella*, *Lelliottia*). This supports the hypothesis that the *B*. *obscurus* symbiont B, also present in *Chrysochus asclepiadeus*, is related to quite generalist gut bacteria, suggesting a loose association with the host. While the *B*. *obscurus* symbiont A is in the same clade with the other subfamily specific Chrysomelidae symbionts, and since it has been detected also in both the Eumolpinae species included in this study, it is probably widespread in the subfamily.

### Microbiota diversity

The comparison of α‐diversity metrics between the specialist and the generalist species included in this study confirms that those usually feeding on several plant families harbour a more diversified microbiota (Fig. 3). The higher microbiota richness of generalist insects has been previously observed comparing different insect orders and broad diet categories (e.g., detritivores vs. herbivores/carnivores) (Colman *et al*., 2012; Yun *et al*., 2014). Also, a study performed on Tephritidae supports this hypothesis (Ventura *et al*., 2018), while other studies on different taxonomic groups do not confirm this pattern (Blankenchip *et al*., 2018; Rothman *et al*., 2020). The higher microbiota diversity observed in generalist insects can be the result of bacteria randomly acquired from the environment, without any specific functional role in the host physiology, or due to the establishment of a diversified microbiota that provides adaptive advantages to the host (i.e., a wider metabolic potential that allows the exploitation of diversified food sources). An exemplar case can be identified in detritivorous insects that harbour one of the richest microbiotas among insects (Colman *et al*., 2012). Detritivorous insects' food source is composed of substrates of different origins that are colonized by a high variety of bacterial taxa, potentially contributing to insect microbiota diversity. On the other hand, detritus includes some of the most difficult molecules to digest (e.g., lignocellulose), thus insects could benefit from the amplified metabolic capabilities supplied by a richer microbiota. Also in the case of phytophagous insects, the higher microbiota richness observed in generalist species can be easily related to the acquisition of different bacteria that are part of the environmental microbial communities (Montagna *et al*., 2015b; Chouaia *et al*., 2019; Hannula *et al*., 2019; Jones *et al*., 2019). The host plant–soil system is one of the major drivers of the phytophagous insect microbiota (Hannula *et al*., 2019), thus feeding on more than one plant species can highly influence insect's microbiota diversity and composition (Jones *et al*., 2019). Secondary metabolites (Zhang *et al*., 2020a; Zhang *et al*., 2020b) and plant defences (Chung *et al*., 2017) often play a fundamental role in these plant–insect–microbiota interactions. Moreover, bacteria colonizing plant surfaces and tissues can be able to degrade the toxic compounds produced by the plant itself (e.g., Shukla and Beran, 2020; Leite‐Mondin *et al*., 2021). So, insects feeding on plants can acquire bacteria that, if established in their microbiota, can provide adaptative advantages. In fact, a richer microbiota determines a wider range of metabolic capabilities that can help the phytophagous insect to overcome the defences of different host plants (e.g., Martinez *et al*., 2019; Santos‐Garcia *et al*., 2020), thus also allowing the expansion of the trophic spectrum. Distinguishing between the processes that shape the microbiota diversity of generalist insects, especially if phytophagous, is quite difficult. Indeed, our results suggest that probably both the random acquisition from the environment and the adaptive advantage of an amplified metabolic potential participate to increase the diversity of the microbiota of generalist species. In this study, the estimated diversity of the microbiota is always higher in generalist species. This is observed both when the diversity value is estimated considering all bacteria (the weight is mostly on rare and low‐abundance species, more likely acquired from the environment; *q* = 0), as well as when the weight is on more common species (considering mainly medium‐high abundance bacteria having a possible functional role; *q* = 1, *q* = 2) (Fig. 3). The importance of the food source in influencing the composition of the microbiota is also highlighted by the higher β‐diversity observed in specialist insects when focusing on rare bacteria (*q* = 0) (Table 2). In fact, the microbiota of each specialist species results simpler than that of each generalist species (lower α‐diversity) but considering together all the species in each of the two groups the overall diversity reaches similar levels (same γ‐diversity). Based on previous results, the overall microbiota diversity of the specialists is mainly due to the high amount of not‐shared bacterial taxa among species (reflected by a high β‐diversity). This can be explained by the acquisition of phylogenetically distant bacteria from the host plant exploited by each insect species, supporting the importance of the food source in influencing the microbiota of phytophagous insects. The higher α‐diversity of the microbiota harboured by generalist species (approximately twice higher than that of specialists) is confirmed also when focusing on dominant bacteria (*q* = 2; Fig. 3). In this last case the difference is probably related to non‐transient bacteria that may have a functional role in insect physiology. These results support the hypothesis that the high bacterial diversity hosted by generalist insects can expand the host metabolic potential enabling the exploitation of different food sources. Further studies, with an increased sample size or focusing on other phytophagous insect groups, are needed to confirm the pattern here observed and to better clarify the most influential causes of this phenomenon.

## Experimental procedures

### Species selection and host plant information

Thirty adult insects, collected from vegetation by sweep net and identified as belonging to 30 different species of Chrysomelidae, have been selected for this study (Table 1). The selection was performed to maximize the taxonomic coverage and the representativeness of the variability in the trophic specialization. The sampling includes representatives of the 10 main subfamilies of Chrysomelidae: Alticinae, Chrysomelinae, Galerucinae, Donacinae, Criocerinae, Cassidinae (including Hispini), Cryptocephalinae (including Clitrini), Eumolpinae, Orsodacninae, Zeugophorinae. The list of plants included in the diet of each Chrysomelidae species was compiled from a database of the host plants of Euro‐Mediterranean Chrysomelidae (Magoga *et al*. in preparation). The trophic spectrum of the selected species ranges from exclusively monophagous species, restricted to feed on a single plant species (e.g., *Chrysocus asclepiadeus* feeds only on *Vincetoxicum hirundinaria*, Apocynaceae), to extremely polyphagous species able to exploit several different food sources (e.g., *Cryptocephalus fulvus* feeds on several plant species belonging to at least 14 different families). To compare the structure and diversity of the microbiota of Chrysomelidae with different breadth of the trophic spectrum, the selected species were divided into trophic classes (generalist and specialist) depending on the number of host plant families: (i) specialist includes species feeding on a single plant family; (ii) generalist includes species exploiting more plant families.

**Table 1:** Information on the analysed samples.

Species	Diet	Subfamily	Date	Country	Latitude	Longitude
*Altica oleracea*	Generalist	Alticinae	08/02/2010	Italy	45.794 N	9.250 E
*Chaetocnema hortensis*	Generalist	Alticinae	08/02/2010	Italy	45.794 N	9.250 E
*Crepidodera fulvicornis*	Generalist	Alticinae	08/02/2010	Italy	45.794 N	9.250 E
*Cassida inopinata*	Specialist	Cassidinae	28/06/2009	Italy	44.517 N	8.819 E
*Hypocassida subferruginea*	Generalist	Cassidinae	14/07/2008	Italy	42.796 N	11.242 E
*Dicladispa testacea*	Specialist	Cassidinae	03/06/2011	Italy	44.195 N	8.281 E
*Hispa atra*	Generalist	Cassidinae	26/06/2011	France	42.512 N	2.124 E
*Clytra quadripunctata*	Generalist	Chryptocephalinae	17/07/2010	Italy	45.941 N	9.416 E
*Cryptocephalus fulvus*	Generalist	Chryptocephalinae	06/04/2010	Italy	40.855 N	12.956 E
*Cryptocephalus loreyi*	Generalist	Chryptocephalinae	17/05/2009	Italy	45.857 N	9.253 E
*Cryptocephalus transcaucasicus*	Generalist	Chryptocephalinae	25/07/2009	Italy	44.702 N	7.142 E
*Labidostomis longimana*	Generalist	Chryptocephalinae	13/07/2010	Italy	45.824 N	9.279 E
*Pachybrachis exclusus*	Specialist	Chryptocephalinae	21/06/2008	Italy	44.056 N	9.832 E
*Smaragdina affinis*	Generalist	Chryptocephalinae	17/05/2009	Italy	45.857 N	9.253 E
*Calligrapha* sp.	Unknown	Chrysomelinae	01/04/2017	USA	32.268 N	110.808 W
*Chrysolina fastuosa*	Generalist	Chrysomelinae	17/05/2009	Italy	45.857 N	9.253 E
*Chrysomela saliceti*	Specialist	Chrysomelinae	21/06/2011	Italy	44.456 N	9.823 E
*Prasocuris phellandrii*	Generalist	Chrysomelinae	24/04/2010	Italy	45.795 N	9.216 E
*Timarcha tenebricosa*	Generalist	Chrysomelinae	04/06/2012	France	43.847 N	6.518 E
*Crioceris paracenthesis*	Specialist	Criocerinae	04/06/2012	Italy	45.894 N	13.551 E
*Lilioceris merdigera*	Generalist	Criocerinae	21/05/2010	Italy	45.794 N	9.250 E
*Donacia obscura*	Specialist	Donacinae	11/05/2011	Italy	44.625 N	9.542 E
*Plateumaris consimilis*	Generalist	Donacinae	07/04/2017	Italy	45.794 N	9.250 E
*Chrysocus asclepiadeus*	Specialist	Eumolpinae	28/06/2010	Italy	45.831 N	9.286 E
*Macrocoma henoni*	Unknown	Eumolpinae	23/05/2013	Morocco	31.150 N	5.393 W
*Exosoma thoracicum*	Generalist	Galerucinae	11/06/2011	Turkey	37.232 N	27.611 E
*Luperus longicornis*	Generalist	Galerucinae	28/06/2010	Italy	45.831 N	9.286 E
*Orsodacne cerasi*	Generalist	Orsodacninae	28/05/2009	Italy	45.893 N	9.281 E
*Orsodacne humeralis*	Generalist	Orsodacninae	21/05/2011	Turkey	39.761 N	27.571 E
*Zeugophora flavicollis*	Specialist	Zeugophorinae	08/07/2011	Italy	45.943 N	9.410 E

Taxonomic (species, families), ecological (diet spectrum width, i.e., specialist or generalist) and collection (date, country, latitude, longitude) information are reported.

**Table 2:** Diversity partitioning.

Group	*q*	α‐diversity	γ‐diversity	β‐diversity
Total dataset	0	35.2	318.4	9.0
	1	1.9	5.5	2.9
	2	1.2	3.0	2.5
Specialists	0	26.8	318.4	11.9
	1	2.7	4.9	1.8
	2	2.0	2.9	1.4
Generalists	0	39.1	318.4	8.1
	1	3.5	6.4	1.8
	2	2.3	3.0	1.3

The three diversity components (α‐diversity, within sample diversity; γ‐diversity, whole group diversity; β‐diversity, among samples diversity) are reported for the whole dataset (total dataset) and for each of the two trophic categories considered (specialists and generalists).

### DNA extraction

DNA was extracted from the whole insect body using the classical phenol–chloroform methods (Doyle and Doyle, 1990) with the following modifications. First, 500 μl of 2% CTAB (2% CTAB, 0.2% ascorbic acid, 1.5% PVP, 1.4 mmol L^−1^ NaCl, 20 mmol L^−1^ EDTA and 100 mmol L^−1^ Tris–HCl, pH 8.0) was added to each sample. Tissues were then disrupted using glass beads (ø 0.1 mm) with the Precellys®24 homogenizer (Bertin Technologies, Montigny‐le‐Bretonneux, France) and incubated at 65°C for 15 min to inactivate nucleases. After centrifugation, the supernatant was incubated overnight with 20 μl of proteinase K (20 mg ml^−1^) at 56°C. To purify the DNA, two phenol–chloroform washes (phenol/chloroform/isoamyl alcohol, 25:24:1, pH 8.0) were performed. DNA was, then, precipitated after addition of 500 μl of isopropanol and incubation for 1 h. Pellet was washed twice with 70% ethanol and eluted in 40 μl of Ultrapure Water (Sigma‐Aldrich, Saint Louis, Missouri, USA). Qubit 4.0 fluorometer (Thermo Fisher Scientific) was used to determine the DNA concentrations. A DNA extraction blank, using the same extraction protocol and molecular biology grade water, was performed as control to monitor environmental contamination.

### Library preparation and sequencing

Two regions of 16S rRNA gene (V1–V2 and V4) were sequenced by means of Ion Torrent platform (Life Technologies). PCR primers 27FYM (Frank *et al*., 2008) and 338R (Amann *et al*., 1990) were used to amplify V1–V2 region, while primers 515F (Caporaso *et al*., 2011), 802R (Claesson *et al*., 2009) and 806R (Caporaso *et al*., 2011) were used for V4 region, in two separated reactions. PCR primers were tailed with two different GC rich sequences enabling barcoding in a second amplification. The first PCR amplification of the V4 region has been performed as reported in Chouaia *et al*. (2019). The first V1–V2 PCR was performed in the same conditions as V4 following 34 cycles of 94°C for 15 s, 60°C for 15 s, 72°C for 15 s and a final extension of 72°C for 2 min. The second PCR amplification was performed in 25 μl volume containing 10 μl HotMasterMix 5Prime 2.5× (Quanta Bio), 1.25 μl EvaGreen™ 20× (Biotium), 1.5 μl barcoded primer (10 μM), 1 μl of the first PCR amplification with the following conditions: 8 cycles of 94°C for 10 s, 60°C for 10 s, 65°C for 40 s and a final extension of 72°C for 3 min. To control for bacterial contaminations, PCR amplifications of the V1–V2 and V4 regions were performed, as previously reported, using as template the DNA extraction blank (i.e., reagents of the used DNA extraction kit) and the PCR reagents. No amplicons were obtained by visualization on 1.5% agarose gel electrophoresis. Furthermore, real‐time PCRs were performed on the DNA extraction blank in order to monitor for contamination and select the appropriate number of the first PCR cycles to avoid the raise of the negative control curves. All the amplicons were checked for their quality and size by agarose gel electrophoresis, quantified with the Qubit™ dsDNA BR Assay Kit in the Qubit 2.0 Fluorometer (Thermo Fisher Scientific) and pooled together in equimolar amounts. The library was purified running it in a precasted E‐Gel® SizeSelect™ (Invitrogen) agarose gel 2% and finally quality checked and quantified with High Sensitivity DNA reagents in the Agilent 2100 Bioanalyzer (Agilent Technologies). For sequencing the library was first subjected to emulsion PCR on the Ion OneTouch™ 2 system using the Ion PGM™ Template Hi‐Q OT2 View (Life Technologies) according to the manufacturer's instructions. Then ion sphere particles (ISPs) were enriched using the E/S module. Resultant live ISPs were loaded and sequenced on an Ion 316 chip (Life Technologies) in the Ion Torrent PGM System.

### Bioinformatic analyses

The bioinformatic analyses were performed using the QIIME2 platform (Bolyen *et al*., 2019). The obtained raw reads for the two 16S rRNA gene regions (V1–V2 and V4) were denoised and taxonomically annotated separately. The DADA2 algorithm (Callahan *et al*., 2016) was used for denoising to obtain an estimation of the actual ASVs present (ASVs) using default parameters (e.g., trunQ = 2, maxEE = 2). The obtained ASVs have been taxonomically annotated with the fit‐classifier‐sklearn method (Pedregosa *et al*., 2011; Bokulich *et al*., 2018) using the release 138 of the SILVA database (Quast *et al*., 2012) as reference for sequences and taxonomy. The naïve Bayes classifiers were trained on the reference sequences trimmed to correspond to the amplified region. To obtain a common phylogeny for the ASVs from the two 16S rRNA regions, the SEPP technique (SATé‐enabled phylogenetic placement; Janssen *et al*., 2018) was applied to place the ASVs on a reference phylogeny based on the release 138 of the SILVA database (Quast *et al*., 2012). In specific cases (possible Enterobacteriaceae primary symbionts) the taxonomic annotation of the ASVs have been checked using the BLAST algorithm (Altschul *et al*., 1990) on the NCBI nt database and confirmed using phylogenetic tree inference.

To infer maximum‐likelihood trees for the phylogenetic placement of putative primary symbionts, 16S rRNA reference sequences for selected genera representative of the Enterobacteriaceae were downloaded from the NCBI nt database. Sequences were aligned using the mafft algorithm v.7.471 (Katoh and Standley, 2013) considering information on the secondary structures of the rRNA. The ML trees have been inferred with iq‐tree v.2.0.3 (Minh *et al*., 2020) using ModelFinder (Kalyaanamoorthy *et al*., 2017) to select the substitution model according with AIC (Akaike, 1973). Ten trees for each amplified region have been inferred to check for concordance of different runs. The same phylogenetic tree inference pipeline has been applied to manually aligned COI sequences of the 30 selected Chrysomelidae species (Magoga *et al*., 2018) (Supplementary Table 3) with the topology constrained to the one published in Nie *et al*. (2020).

The microbiota diversity analyses were performed with a sample size and coverage‐based integrations of interpolation (rarefaction) and extrapolation (prediction) of the Hill numbers (Hill, 1973; Alberdi and Gilbert, 2019; Roswell *et al*., 2021) using the R packages iNEXT and iNextPD (Chao *et al*., 2014, 2015; Hsieh *et al*., 2016). The computation of Hill numbers was performed for three increasing values of the order parameter *q*, corresponding to increasing weight on the species abundance (or any other taxonomic level considered) and also to different well‐known diversity and phylogenetic diversity indices: *q* = 0, counting mainly the rare species (those with low abundances), corresponds to richness (McIntosh, 1967) and Faith's Phylogenetic Diversity (Faith, 1992); *q* = 1, counting mainly the common species (those with medium‐high abundances), corresponds to the exponential of Shannon index (Shannon, 1948) and Allen's Phylogenetic entropy (Allen *et al*., 2009); *q* = 2, counting mainly the dominant species (those with very high abundances), corresponds to the inverse of Simpson index (Simpson, 1949) and Rao's quadratic entropy (Rao, 1982). This explicit parametrization is particularly useful to test our hypothesis, since we can assume that symbionts with a functional role are present at high abundances (*q* = 1, *q* = 2) while the bacteria acquired from the environment have usually low abundances, so can be considered rare species (*q* = 0). The hilldiv R package (Alberdi and Gilbert, 2021) was used to partition the diversity in its components: α‐diversity (diversity at the sample level), γ‐diversity (total diversity in the selected group) and β‐diversity (γ‐diversity/α‐diversity, corresponding to the among sample component of the total diversity). Comparing diversity partitioning between the two trophic groups considered in this study (specialists and generalists) allows us to understand which component is most influential in determining the different diversity estimates. The R package phytools (Revell, 2012) was used for ML ancestral state reconstruction (Revell, 2013) of the microbiota diversity estimates along the insect phylogenetic tree and to compute phylogenetic signals (Blomberg *et al*., 2003; Ives *et al*., 2007).

## Supporting information


**Supplementary Fig. 1.** Bacteria abundance in single marker datasets. Heatmap representing the abundance of bacterial taxa (classes, families, genera) present in the single marker datasets (V4 and V1‐V2). In the genera heatmap only the 50 most abundant genera are shown. Colour intensity is proportional to the normalized relative abundance of the bacterial taxa.

**Supplementary Fig. 2.** Microbiota diversity estimates inferred on the total dataset (V1‐V2 and V4), V1‐V2 and V4 regions of the 16S rRNA. Sample‐based rarefaction/extrapolation curves of the Hill numbers estimated for three values of the order parameter (q = 0, q = 1, q = 2). The x‐axis represents increasing sampling and the y‐axis represents the Hill number estimates, 95% confidence interval is also reported. As reported in the legend, colours correspond to the trophic category (specialist or generalist) and line type to the methodological approach (interpolation or extrapolation). a) Global dataset (V1‐V2 and V4 regions of the16S rRNA). b) V1‐V2 region of the 16S rRNA. c) V4 region of the 16S rRNA.

**Supplementary Fig. 3.** Maximum likelihood phylogenetic trees. Sequences obtained from the NCBI database report the accession numbers while sequences produced in this study are highlighted in bold. a) Tree obtained from sequences of the V1‐V2 region of the 16S rRNA. b) Tree obtained from sequences of the V4 region of the 16S rRNA.

**Supplementary Fig. 4.** Microbiota diversity of specialist and generalist Chrysomelidae defined using the plant taxonomic level of genus (specialists feed on plants all belonging to the same genus, generalists feed on plants belonging to different genera). Coverage based rarefaction/extrapolation curves of the Hill numbers estimated for three values of the order parameter (q = 0, q = 1, q = 2). The x‐axis represents the coverage (that estimates the completeness of the sampling) and the y‐axis represents the Hill number estimates, 95% confidence interval is also reported. As reported in the legend, colours correspond to the trophic category (specialist or generalist) and line type to the methodological approach (interpolation or extrapolation).

**Supplementary Table 1.** Primary symbionts relative abundance.
**Supplementary Table 2**. Blast search results on NCBI nt database.
**Supplementary Table 3**. Accession numbers of COI sequences.

## References

[emi15847-bib-0001] Akaike, H. (1973) Information theory and an extension of the maximum likelihood principle. In Proceedings of the Second International Symposium on Information Theory, Petrov, B.N. , and Csàki, F. (eds). Akadémiai Kiadò: Budapest, pp. 267–281.

[emi15847-bib-0002] Alberdi, A. , and Gilbert, M.T.P. (2019) A guide to the application of Hill numbers to DNA‐based diversity analyses. Mol Ecol Resour 19: 804–817. 30947383 10.1111/1755-0998.13014

[emi15847-bib-0003] Alberdi, A. , and Gilbert, M.T.P. (2021) hilldiv: an R package for the integral analysis of diversity based on Hill numbers. Biorxiv. 545665. 10.1101/545665

[emi15847-bib-0004] Ali, H. , Muhammad, A. , Sanda, N.B. , Huang, Y. , and Hou, Y. (2019) Pyrosequencing uncovers a shift in bacterial communities across life stages of *Octodonta nipae* (Coleoptera: Chrysomelidae). Front Microbiol 10: 466. 30930872 10.3389/fmicb.2019.00466 PMC6424052

[emi15847-bib-0005] Allen, B. , Kon, M. , and Bar‐Yam, Y. (2009) A new phylogenetic diversity measure generalizing the Shannon index and its application to phyllostomid bats. Am Nat 174: 236–243. 19548837 10.1086/600101

[emi15847-bib-0006] Altschul, S.F. , Gish, W. , Miller, W. , Myers, E.W. , and Lipman, D.J. (1990) Basic local alignment search tool. J Mol Biol 215: 403–410. 2231712 10.1016/S0022-2836(05)80360-2

[emi15847-bib-0007] Amann, R.I. , Binder, B.J. , Olson, R.J. , Chisholm, S.W. , Devereux, R. , and Stahl, D.A. (1990) Combination of 16S rRNA‐targeted oligonucleotide probes with flow cytometry for analyzing mixed microbial populations. Appl Environ Microbiol 56: 1919–1925. 2200342 10.1128/aem.56.6.1919-1925.1990 PMC184531

[emi15847-bib-0008] Ankrah, N.Y.D. , Chouaia, B. , and Douglas, A.E. (2018) The cost of metabolic interactions in symbioses between insects and bacteria with reduced genomes. mBio 9: e01433–18. 10.1128/mBio.01433-18 30254121 PMC6156193

[emi15847-bib-0009] Becker, M. (1994) The female organs of symbiont transmission in the Eumolpinae. In Novel Aspects of the Biology of Chrysomelidae. Series Entomologica, Vol. 50, Jolivet, P.H. , Cox, M.L. , and Petitpierre, E. (eds). Dordrecht: Springer, pp. 363–370.

[emi15847-bib-0010] Blankenchip, C.L. , Michels, D.E. , Braker, H.E. , and Goffredi, S.K. (2018) Diet breadth and exploitation of exotic plants shift the core microbiome of *Cephaloleia*, a group of tropical herbivorous beetles. PeerJ 6: e4793. 29785353 10.7717/peerj.4793 PMC5960584

[emi15847-bib-0011] Blomberg, S.P. , Garland, T., Jr. , and Ives, A.R. (2003) Testing for phylogenetic signal in comparative data: behavioral traits are more labile. Evolution 57: 717–745. 12778543 10.1111/j.0014-3820.2003.tb00285.x

[emi15847-bib-0012] Bokulich, N.A. , Kaehler, B.D. , Rideout, J.R. , Dillon, M. , Bolyen, E. , Knight, R. , *et al*. (2018) Optimizing taxonomic classification of marker‐gene amplicon sequences with QIIME 2's q2‐feature‐classifier plugin. Microbiome 6: 90. 29773078 10.1186/s40168-018-0470-z PMC5956843

[emi15847-bib-0014] Bolyen, E. , Rideout, J.R. , Dillon, M.R. , Bokulich, N.A. , Abnet, C.C. , Al‐Ghalith, G.A. , *et al*. (2019) Reproducible, interactive, scalable and extensible microbiome data science using QIIME 2. Nat Biotechnol 37: 852–857. 31341288 10.1038/s41587-019-0209-9 PMC7015180

[emi15847-bib-0015] Boscaro, V. , Kolisko, M. , Felletti, M. , Vannini, C. , Lynn, D.H. , and Keeling, P.J. (2017) Parallel genome reduction in symbionts descended from closely related free‐living bacteria. Nat Ecol Evol 1: 1160–1167. 29046583 10.1038/s41559-017-0237-0

[emi15847-bib-0016] Brady, C. , Cleenwerck, I. , Venter, S. , Coutinho, T. , and De Vos, P. (2013) Taxonomic evaluation of the genus Enterobacter based on multilocus sequence analysis (MLSA): proposal to reclassify *E*. *nimipressuralis* and *E*. *amnigenus* into *Lelliottia* gen. nov. as *Lelliottia nimipressuralis* comb. nov. and *Lelliottia amnigena* comb. nov., respectively, *E*. *gergoviae* and *E*. *pyrinus* into *Pluralibacter* gen. nov. as *Pluralibacter gergoviae* comb. nov. and *Pluralibacter pyrinus* comb. nov., respectively, *E*. *cowanii*, *E*. *radicincitans*, *E*. *oryzae* and *E*. *arachidis* into *Kosakonia* gen. nov. as *Kosakonia cowanii* comb. nov., *Kosakonia radicincitans* comb. nov., *Kosakonia oryzae* comb. nov. and *Kosakonia arachidis* comb. nov., respectively, and *E*. *turicensis*, *E*. *helveticus* and *E*. *pulveris* into *Cronobacter* as *Cronobacter zurichensis* nom. nov., *Cronobacter helveticus* comb. nov. and *Cronobacter pulveris* comb. nov., respectively, and emended description of the genera *Enterobacter* and *Cronobacter* . Syst Appl Microbiol 36: 309–319. 23632228 10.1016/j.syapm.2013.03.005

[emi15847-bib-0017] Brisse, S. , Grimont, F. , and Grimont, P.A.D. (2006) The genus Klebsiella. In The Prokaryotes: Proteobacteria: Gamma Subclass, Vol. 6, Dworkin, M. , Falkow, S. , Rosenberg, E. , Schleifer, K.H. , and Stackebrandt, E. (eds). New York: Springer, pp. 159–196.

[emi15847-bib-0018] Buchner, P. (1965) Endosymbiosis of Animals with Plant Microorganisms. New York, USA: Interscience Publishers.

[emi15847-bib-0019] Callahan, B.J. , McMurdie, P.J. , Rosen, M.J. , Han, A.W. , Johnson, A.J.A. , and Holmes, S.P. (2016) DADA2: high‐resolution sample inference from Illumina amplicon data. Nat Methods 13: 581–583. 27214047 10.1038/nmeth.3869 PMC4927377

[emi15847-bib-0020] Caporaso, J.G. , Lauber, C.L. , Walters, W.A. , Berg‐Lyons, D. , Lozupone, C.A. , Turnbaugh, P.J. , *et al*. (2011) Global patterns of 16S rRNA diversity at a depth of millions of sequences per sample. Proc Natl Acad Sci U S A 108: 4516–4522. 20534432 10.1073/pnas.1000080107 PMC3063599

[emi15847-bib-0021] Cardoso, A. , and Gómez‐Zurita, J. (2020) Food resource sharing of alder leaf beetle specialists (Coleoptera: Chrysomelidae) as potential insect–plant interface for horizontal transmission of endosymbionts. Environ Entomol 49: 1402–1414. 33315074 10.1093/ee/nvaa111 PMC7734963

[emi15847-bib-0022] Ceja‐Navarro, J.A. , Vega, F.E. , Karaoz, U. , Hao, Z. , Jenkins, S. , Lim, H.C. , *et al*. (2015) Gut microbiota mediate caffeine detoxification in the primary insect pest of coffee. Nat Commun 6: 7618. 26173063 10.1038/ncomms8618 PMC4510693

[emi15847-bib-0134] Chao, A. , Chiu, C.‐H. , Hsieh, T.C. , Davis, T. , Nipperess, D.A. , and Faith, D.P. (2015). Rarefaction and extrapolation of phylogenetic diversity. Method Ecol Evol 6: 380–388. 10.1111/2041-210x.12247

[emi15847-bib-0135] Chao, A. , Gotelli, N.J. , Hsieh, T.C. , Sander, E.L. , Ma, K.H. , Colwell, R.K. , and Ellison, A.M. (2014). Rarefaction and extrapolation with Hill numbers: a framework for sampling and estimation in species diversity studies. Ecol Monogr 84: 45–67. 10.1890/13-0133.1

[emi15847-bib-0023] Chen, G. , Fang, E. , Mak, S. , and Xiong, L. (2021) Diet affects the composition and diversity of the mammalian gut microbiota. JEMI 26: 1–11.

[emi15847-bib-0024] Chen, Z. , Hui, P.C. , Hui, M. , Yeoh, Y.K. , Wong, P.Y. , Chan, M.C. , *et al*. (2019) Impact of preservation method and 16S rRNA hypervariable region on gut microbiota profiling. Msystems 4: e00271–18. 10.1128/mSystems.00271-18. 30834331 PMC6392095

[emi15847-bib-0025] Chouaia, B. , Goda, N. , Mazza, G. , Alali, S. , Florian, F. , Gionechetti, F. , *et al*. (2019) Developmental stages and gut microenvironments influence gut microbiota dynamics in the invasive beetle *Popillia japonica* Newman (Coleoptera: Scarabaeidae). Environ Microbiol 21: 4343–4359. 31502415 10.1111/1462-2920.14797

[emi15847-bib-0026] Chrostek, E. , Pelz‐Stelinski, K. , Hurst, G.D. , and Hughes, G.L. (2017) Horizontal transmission of intracellular insect symbionts via plants. Front Microbiol 8: 2237. 29234308 10.3389/fmicb.2017.02237 PMC5712413

[emi15847-bib-0027] Chung, S.H. , Rosa, C. , Scully, E.D. , Peiffer, M. , Tooker, J.F. , Hoover, K. , *et al*. (2013) Herbivore exploits orally secreted bacteria to suppress plant defenses. Proc Natl Acad Sci USA 110: 733–15733. 10.1073/pnas.1308867110 PMC3785742 24019469

[emi15847-bib-0028] Chung, S.H. , Scully, E.D. , Peiffer, M. , Geib, S.M. , Rosa, C. , Hoover, K. , and Felton, G.W. (2017) Host plant species determines symbiotic bacterial community mediating suppression of plant defenses. Sci Rep 7: 1–13. 28045052 10.1038/srep39690 PMC5206732

[emi15847-bib-0029] Claesson, M.J. , O'Sullivan, O. , Wang, Q. , Nikkilä, J. , Marchesi, J.R. , Smidt, H. , *et al*. (2009) Comparative analysis of pyrosequencing and a phylogenetic microarray for exploring microbial community structures in the human distal intestine. PloS One 4: e6669. 19693277 10.1371/journal.pone.0006669 PMC2725325

[emi15847-bib-0030] Clark, T.L. , Meinke, L.J. , Skoda, S.R. , and Foster, J.E. (2001) Occurrence of *Wolbachia* in selected diabroticite (Coleoptera: Chrysomelidae) beetles. Ann Entomol Soc Am 94: 877–885.

[emi15847-bib-0031] Clay, K. (2014) Defensive symbiosis: a microbial perspective. Funct Ecol 28: 293–298.

[emi15847-bib-0032] Colman, D.R. , Toolson, E.C. , and Takacs‐Vesbach, C.D. (2012) Do diet and taxonomy influence insect gut bacterial communities? Mol Ecol 21: 5124–5137. 22978555 10.1111/j.1365-294X.2012.05752.x

[emi15847-bib-0033] Correa, C.C. , and Ballard, J.W.O. (2016) *Wolbachia* associations with insects: winning or losing against a master manipulator. Front Ecol Evol 3: 153.

[emi15847-bib-0034] Dantur, K.I. , Enrique, R. , Welin, B. , and Castagnaro, A.P. (2015) Isolation of cellulolytic bacteria from the intestine of *Diatraea saccharalis* larvae and evaluation of their capacity to degrade sugarcane biomass. Amb Express 5: 15. 25852992 10.1186/s13568-015-0101-z PMC4385043

[emi15847-bib-0035] Dillon, R.J. , Vennard, C.T. , and Charnley, A.K. (2002) A note: gut bacteria produce components of a locust cohesion pheromone. J Appl Microbiol 92: 759–763. 11966918 10.1046/j.1365-2672.2002.01581.x

[emi15847-bib-0036] Douglas, A.E. (2009) The microbial dimension in insect nutritional ecology. Funct Ecol 23: 38–47.

[emi15847-bib-0037] Douglas, A.E. (2015) Multiorganismal insects: diversity and function of resident microorganisms. Annu Rev Entomol 60: 17–34. 25341109 10.1146/annurev-ento-010814-020822 PMC4465791

[emi15847-bib-0038] Doyle, J.J. , and Doyle, J.L. (1990) Isolation of plant DNA from fresh tissue. Focus 12: 39–40.

[emi15847-bib-0039] Engel, P. , and Moran, N.A. (2013) The gut microbiota of insects–diversity in structure and function. FEMS Microbiol Rev 37: 699–735. 23692388 10.1111/1574-6976.12025

[emi15847-bib-0040] Engelstädter, J. , and Hurst, G.D. (2009) The ecology and evolution of microbes that manipulate host reproduction. Annu Rev Ecol Evol Syst 40: 127–149.

[emi15847-bib-0041] Faith, D.P. (1992) Conservation evaluation and phylogenetic diversity. Biol Conserv 61: 1–10.

[emi15847-bib-0042] Frago, E. , Zytynska, S.E. , and Fatouros, N.E. (2020) Microbial symbionts of herbivorous species across the insect tree. In Advances in Insect Physiology: Mechanisms Underlying Microbial Symbiosis, Vol. 58, Oliver, K.M. , and Russel, J.A. (eds). London, UK: Academic Press, pp. 111–159.

[emi15847-bib-0043] Frank, J.A. , Reich, C.I. , Sharma, S. , Weisbaum, J.S. , Wilson, B.A. , and Olsen, G.J. (2008) Critical evaluation of two primers commonly used for amplification of bacterial 16S rRNA genes. Appl Environ Microbiol 74: 2461–2470. 18296538 10.1128/AEM.02272-07 PMC2293150

[emi15847-bib-0044] Fukumori, K. , Koga, R. , Nikoh, N. , and Fukatsu, T. (2017) Symbiotic bacteria associated with gut symbiotic organs and female genital accessory organs of the leaf beetle *Bromius obscurus* (Coleoptera: Chrysomelidae). Appl Entomol Zool 52: 589–598.

[emi15847-bib-0045] Giron, D. , Dedeine, F. , Dubreuil, G. , Huguet, E. , Mouton, L. , Outreman, Y. , *et al*. (2017) Influence of microbial symbionts on plant–insect interactions. In Advances in Botanical Research, Vol. 81, Sauvion, N. , Thiéry, D. , and Calatayud, P. (eds). London, UK: Academic Press, pp. 225–257.

[emi15847-bib-0046] Gómez‐Zurita, J. (2019) Assessment of the role of *Wolbachia* in mtDNA paraphyly and the evolution of unisexuality in Calligrapha (Coleoptera: Chrysomelidae). Ecol Evol 9: 11198–11214. 31641465 10.1002/ece3.5621 PMC6802014

[emi15847-bib-0048] Hannula, S.E. , Zhu, F. , Heinen, R. , and Bezemer, T.M. (2019) Foliar‐feeding insects acquire microbiomes from the soil rather than the host plant. Nat Commun 10: 1–9. 30890706 10.1038/s41467-019-09284-w PMC6425034

[emi15847-bib-0049] Hansen, A.K. , and Moran, N.A. (2014) The impact of microbial symbionts on host plant utilization by herbivorous insects. Mol Ecol 23: 1473–1496. 23952067 10.1111/mec.12421

[emi15847-bib-0051] Harris, H.L. , Brennan, L.J. , Keddie, B.A. , and Braig, H.R. (2010) Bacterial symbionts in insects: balancing life and death. Symbiosis 51: 37–53.

[emi15847-bib-0053] Hill, M.O. (1973) Diversity and evenness: a unifying notation and its consequences. Ecology 54: 427–432.

[emi15847-bib-0133] Hsieh, T.C. , Ma, K.H. , and Chao, A. (2016). iNEXT: an R package for rarefaction and extrapolation of species diversity (Hill numbers). Method Ecol Evol 7: 1451–1456. 10.1111/2041-210x.12613

[emi15847-bib-0054] Huang, S. , Sheng, P. , and Zhang, H. (2012) Isolation and identification of cellulolytic bacteria from the gut of *Holotrichia parallela* larvae (Coleoptera: Scarabaeidae). Int J Mol Sci 13: 2563–2577. 22489111 10.3390/ijms13032563 PMC3317674

[emi15847-bib-0055] Hurst, G.D. , and Frost, C.L. (2015) Reproductive parasitism: maternally inherited symbionts in a biparental world. In Cold Spring Harbor Perspectives in Biology, Rice, W.R. , and Gavrilets, S. (eds). New York, NY: Cold Spring Harbor Laboratory Press, p. a017699. 10.1101/cshperspect.a017699 PMC4448626 25934011

[emi15847-bib-0056] Ives, A.R. , Midford, P.E. , and Garland, T., Jr. (2007) Within‐species variation and measurement error in phylogenetic comparative methods. Syst biol 56: 252–270. 17464881 10.1080/10635150701313830

[emi15847-bib-0057] Janssen, S. , McDonald, D. , Gonzalez, A. , Navas‐Molina, J.A. , Jiang, L. , Xu, Z.Z. , *et al*. (2018) Phylogenetic placement of exact amplicon sequences improves associations with clinical information. Msystems 3: e00021–18. 29719869 10.1128/mSystems.00021-18 PMC5904434

[emi15847-bib-0058] Jones, A.G. , Mason, C.J. , Felton, G.W. , and Hoover, K. (2019) Host plant and population source drive diversity of microbial gut communities in two polyphagous insects. Sci Rep 9: 1–11. 30808905 10.1038/s41598-019-39163-9 PMC6391413

[emi15847-bib-0059] Kajtoch, L. , and Kotásková, N. (2018) Current state of knowledge on *Wolbachia* infection among Coleoptera: a systematic review. PeerJ 6: e4471. 29568706 10.7717/peerj.4471 PMC5846457

[emi15847-bib-0060] Kalyaanamoorthy, S. , Minh, B.Q. , Wong, T.K.F. , von Haeseler, A. , and Jermiin, L.S. (2017) ModelFinder: fast model selection for accurate phylogenetic estimates. Nat Methods 14: 587–589. 28481363 10.1038/nmeth.4285 PMC5453245

[emi15847-bib-0061] Kartzinel, T.R. , Hsing, J.C. , Musili, P.M. , Brown, B.R. , and Pringle, R.M. (2019) Covariation of diet and gut microbiome in African megafauna. Proc Natl Acad Sci U S A 116: 23588–23593. 31685619 10.1073/pnas.1905666116 PMC6876249

[emi15847-bib-0062] Katoh, K. , and Standley, D.M. (2013) MAFFT multiple sequence alignment software version 7: improvements in performance and usability. Mol Biol Evol 30: 772–780. 23329690 10.1093/molbev/mst010 PMC3603318

[emi15847-bib-0063] Keller, G.P. , Windsor, D.M. , Saucedo, J.M. , and Werrin, J.H. (2004) Reproductive effects and geographical distributions of two *Wolbachia* strains infecting the Neotropical beetle, *Chelymorpha alternans* Boh. (Chrysomelidae, Cassidinae). Mol Ecol 13: 2405–2420. 15245413 10.1111/j.1365-294X.2004.02213.x

[emi15847-bib-0064] Kelley, S.T. , and Dobler, S. (2011) Comparative analysis of microbial diversity in *Longitarsus* flea beetles (Coleoptera: Chrysomelidae). Genetica 139: 541–550. 20844936 10.1007/s10709-010-9498-0

[emi15847-bib-0065] Kikuchi, Y. , Hayatsu, M. , Hosokawa, T. , Nagayama, A. , Tago, K. , and Fukatsu, T. (2012) Symbiont‐mediated insecticide resistance. Proc Natl Acad Sci U S A 109: 8618–8622. 22529384 10.1073/pnas.1200231109 PMC3365206

[emi15847-bib-0067] Kleinschmidt, B. , and Kölsch, G. (2011) Adopting bacteria in order to adapt to water – how reed beetles colonized the wetlands (Coleoptera, Chrysomelidae, Donaciinae). Insects 2: 540–554. 26467833 10.3390/insects2040540 PMC4553447

[emi15847-bib-0068] Kolasa, M. , Montagna, M. , Mereghetti, V. , Kubisz, D. , Mazur, M.A. , and Kajtoch, L. (2017) Preliminary evidence of the horizontal transmission of *Wolbachia* between *Crioceris* leaf beetles (Coleoptera: Chrysomelidae) and their *Asparagus* host plants. Eur J Entomol 114: 446–454.

[emi15847-bib-0069] Kolasa, M. , Scibior, R. , Mazur, M.A. , Kubisz, D. , Dudek, K. , and Kajtoch, L. (2019) How hosts taxonomy, trophy, and endosymbionts shape microbiome diversity in beetles. Microb Ecol 78: 995–1013. 30915518 10.1007/s00248-019-01358-y PMC6842344

[emi15847-bib-0070] Kölsch, G. , Matz‐Grund, C. , and Pedersen, B. (2009) Ultrastructural and molecular characterization of endosymbionts of the reed beetle genus *Macroplea* (Chrysomelidae, Donaciinae), and proposal of ‘*Candidatus* Macropleicola appendiculatae’ and ‘*Candidatus* Macropleicola muticae’. J Microbiol 55: 1250–1260. 10.1139/w09-085 19940933

[emi15847-bib-0071] Kölsch, G. , and Pedersen, B.V. (2010) Can the tight co‐speciation between reed beetles (Col., Chrysomelidae, Donaciinae) and their bacterial endosymbionts, which provide cocoon material, clarify the deeper phylogeny of the hosts? Mol Phylogenet Evol 54: 810–821. 19900566 10.1016/j.ympev.2009.10.038

[emi15847-bib-0072] Kölsch, G. , and Synefiaridou, D. (2012) Shared ancestry of symbionts? Sagrinae and Donaciinae (Coleoptera, Chrysomelidae) harbor similar bacteria. Insects 3: 473–491. 26466539 10.3390/insects3020473 PMC4553606

[emi15847-bib-0073] Kondo, N.I. , Tuda, M. , Toquenaga, Y. , Lan, Y.C. , Buranapanichpan, S. , Horng, S.B. , *et al*. (2011) *Wolbachia* infections in world populations of bean beetles (Coleoptera: Chrysomelidae: Bruchinae) infesting cultivated and wild legumes. Zool Sci 28: 501–508. 10.2108/zsj.28.501 21728798

[emi15847-bib-0074] Krawczyk, K. , Szymanczyk, M. , and Obrepalska‐Steplowska, A. (2015) Prevalence of endosymbionts in Polish populations of *Leptinotarsa decemlineata* (Coleoptera: Chrysomelidae). J Insect Sci 15: 1–6. 26206894 10.1093/jisesa/iev085 PMC4672246

[emi15847-bib-0075] Larracuente, A.M. , and Meller, V.H. (2016) Host–symbiont interactions: male‐killers exposed. Curr Biol 26: R429–R431. 27218854 10.1016/j.cub.2016.03.057

[emi15847-bib-0076] Latorre, A. , and Manzano‐Marín, A. (2017) Dissecting genome reduction and trait loss in insect endosymbionts. Ann N Y Acad Sci 1389: 52–75. 27723934 10.1111/nyas.13222

[emi15847-bib-0077] Leite‐Mondin, M. , DiLegge, M.J. , Manter, D.K. , Weir, T.L. , Silva‐Filho, M.C. , and Vivanco, J.M. (2021) The gut microbiota composition of *Trichoplusia ni* is altered by diet and may influence its polyphagous behavior. Sci Rep 11: 1–16. 33707556 10.1038/s41598-021-85057-0 PMC7970945

[emi15847-bib-0078] Leschen, R.A.B. , and Beutel, R.G. (2014) Handbook of Zoology, Band 4: Arthropoda: Insecta, Teilband/Part 40: Coleoptera, Beetles, Morphology and Systematics (Phytophaga), Vol. 3. Berlin: Walter de Gruyter.

[emi15847-bib-0079] Ludwick, D.C. , Ericsson, A.C. , Meihls, L.N. , Gregory, M.L. , Finke, D.L. , Coudron, T.A. , *et al*. (2019) Survey of bacteria associated with western corn rootworm life stages reveals no difference between insects reared in different soils. Sci Rep 9: 1–11. 31653954 10.1038/s41598-019-51870-x PMC6814711

[emi15847-bib-0080] Magoga, G. , Sahin, D.C. , Fontaneto, D. , and Montagna, M. (2018) Barcoding of Chrysomelidae of Euro‐Mediterranean area: efficiency and problematic species. Sci Rep 8: 1–9. 30194432 10.1038/s41598-018-31545-9 PMC6128942

[emi15847-bib-0081] Mann, J.S. , and Crowson, R.A. (1983) On the occurrence of mid‐gut caeca, and organs of symbiont transmission, in leaf‐beetles (Coleoptera: Chrysomelidae). Coleopt Bull 37: 1–15.

[emi15847-bib-0082] Martinez, A.J. , Onchuru, T.O. , Ingham, C.S. , Sandoval‐Calderón, M. , Salem, H. , Deckert, J. , and Kaltenpoth, M. (2019) Angiosperm to gymnosperm host‐plant switch entails shifts in microbiota of the Welwitschia bug, *Probergrothius angolensis* (Distant, 1902). Mol Ecol 28: 5172–5187. 31638716 10.1111/mec.15281

[emi15847-bib-0083] Mason, C.J. (2020) Complex relationships at the intersection of insect gut microbiomes and plant defenses. J Chem Ecol 46: 793–807. 32537721 10.1007/s10886-020-01187-1

[emi15847-bib-0084] Mason, C.J. , Jones, A.G. , and Felton, G.W. (2019) Co‐option of microbial associates by insects and their impact on plant–folivore interactions. Plant Cell Environ 42: 1078–1086. 30151965 10.1111/pce.13430

[emi15847-bib-0085] McIntosh, R.P. (1967) An index of diversity and the relation of certain concepts to diversity. Ecol 48: 392–404.

[emi15847-bib-0086] Minh, B.Q. , Schmidt, H.A. , Chernomor, O. , Schrempf, D. , Woodhams, M.D. , Von Haeseler, A. , and Lanfear, R. (2020) IQ‐TREE 2: new models and efficient methods for phylogenetic inference in the genomic era. Mol Biol Evol 37: 1530–1534. 32011700 10.1093/molbev/msaa015 PMC7182206

[emi15847-bib-0087] Miyashita, A. , Hirai, Y. , Sekimizu, K. , and Kaito, C. (2015) Antibiotic‐producing bacteria from stag beetle mycangia. Drug Discov Ther 9: 33–37. 25639488 10.5582/ddt.2015.01000

[emi15847-bib-0088] Mohammed, W.S. , Ziganshina, E.E. , Shagimardanova, E.I. , Gogoleva, N.E. , and Ziganshin, A.M. (2018) Comparison of intestinal bacterial and fungal communities across various xylophagous beetle larvae (Coleoptera: Cerambycidae). Sci Rep 8: 1–12. 29968731 10.1038/s41598-018-27342-z PMC6030058

[emi15847-bib-0089] Montagna, M. , Chouaia, B. , Mazza, G. , Prosdocimi, E.M. , Crotti, E. , Mereghetti, V. , *et al*. (2015b) Effects of the diet on the microbiota of the red palm weevil (Coleoptera: Dryophthoridae). PLoS One 10: e0117439. 25635833 10.1371/journal.pone.0117439 PMC4311986

[emi15847-bib-0090] Montagna, M. , Chouaia, B. , Sacchi, L. , Porretta, D. , Martin, E. , Giorgi, A. , *et al*. (2014) A new strain of *Wolbachia* in an Alpine population of the viviparous *Oreina cacaliae* (Coleoptera: Chrysomelidae). Environ Entomol 43: 913–922. 25182613 10.1603/EN13228

[emi15847-bib-0091] Montagna, M. , Gómez‐Zurita, J. , Giorgi, A. , Epis, S. , Lozzia, G. , and Bandi, C. (2015a) Metamicrobiomics in herbivore beetles of the genus *Cryptocephalus* (Chrysomelidae): toward the understanding of ecological determinants in insect symbiosis. Insect Sci 22: 340–352. 24871104 10.1111/1744-7917.12143

[emi15847-bib-0092] Moore, E.B. , Tindall, B. , Martins Dos Santos, V.A.P. , Pieper, D. , Ramos, J.L. , and Palleroni, N. (2006) Nonmedical: Pseudomonas. In The Prokaryotes: Proteobacteria: Gamma Subclass, Vol. 6, Dworkin, M. , Falkow, S. , Rosenberg, E. , Schleifer, K.H. , and Stackebrandt, E. (eds). New York: Springer, pp. 646–703.

[emi15847-bib-0095] Muratoglu, H. , Demirbag, Z. , and Sezen, K. (2011) The first investigation of the diversity of bacteria associated with *Leptinotarsa decemlineata* (Coleoptera: Chrysomelidae). Biologia 66: 288–293.

[emi15847-bib-0096] Muturi, E.J. , Dunlap, C. , Ramirez, J.L. , Rooney, A.P. , and Kim, C.H. (2019) Host blood‐meal source has a strong impact on gut microbiota of *Aedes aegypti* . FEMS Microbiol Ecol 95: fiy213. 10.1093/femsec/fiy213 30357406

[emi15847-bib-0097] Nie, R.E. , Andújar, C. , Gómez‐Rodríguez, C. , Bai, M. , Xue, H.J. , Tang, M. , *et al*. (2020) The phylogeny of leaf beetles (Chrysomelidae) inferred from mitochondrial genomes. Syst Entomol 45: 188–204.

[emi15847-bib-0098] Pedregosa, F. , Varoquaux, G. , Gramfort, A. , Michel, V. , Thirion, B. , Grisel, O. , *et al*. (2011) Scikit‐learn: machine learning in python. J Mach Learn Res 12: 2825–2830.

[emi15847-bib-0099] Pérez‐Cobas, A.E. , Maiques, E. , Angelova, A. , Carrasco, P. , Moya, A. , and Latorre, A. (2015) Diet shapes the gut microbiota of the omnivorous cockroach *Blattella germanica* . FEMS Microbiol Ecol 91: fiv022. 25764470 10.1093/femsec/fiv022

[emi15847-bib-0100] Piel, J. , Höfer, I. , and Hui, D. (2004) Evidence for a symbiosis Island involved in horizontal acquisition of pederin biosynthetic capabilities by the bacterial symbiont of *Paederus fuscipes* beetles. J Bacteriol 186: 1280–1286. 14973122 10.1128/JB.186.5.1280-1286.2004 PMC344417

[emi15847-bib-0101] Pietri, J.E. , DeBruhl, H. , and Sullivan, W. (2016) The rich somatic life of *Wolbachia* . Microbiologyopen 5: 923–936. 27461737 10.1002/mbo3.390 PMC5221451

[emi15847-bib-0102] Quast, C. , Pruesse, E. , Yilmaz, P. , Gerken, J. , Schweer, T. , Yarza, P. , *et al*. (2012) The SILVA ribosomal RNA gene database project: improved data processing and web‐based tools. Nucleic Acids Res 41: D590–D596. 23193283 10.1093/nar/gks1219 PMC3531112

[emi15847-bib-0103] Rao, C.R. (1982) Diversity and dissimilarity coefficients: a unified approach. Theor Popul Biol 21: 24–43.

[emi15847-bib-0104] Rasgon, J.L. , Gamston, C.E. , and Ren, X. (2006) Survival of *Wolbachia pipientis* in cell‐free medium. Appl Environ Microbiol 72: 6934–6937. 16950898 10.1128/AEM.01673-06 PMC1636208

[emi15847-bib-0105] Reis, F. , Kirsch, R. , Pauchet, Y. , Bauer, E. , Bilz, L.C. , Fukumori, K. , *et al*. (2020) Bacterial symbionts support larval sap feeding and adult folivory in (semi‐) aquatic reed beetles. Nat Commun 11: 1–15. 32528063 10.1038/s41467-020-16687-7 PMC7289800

[emi15847-bib-0106] Revell, L.J. (2012) phytools: An R package for phylogenetic comparative biology (and other things). Methods Ecol Evol 3: 217–223.

[emi15847-bib-0107] Revell, L.J. (2013) Two new graphical methods for mapping trait evolution on phylogenies. Methods Ecol Evol 4: 754–759.

[emi15847-bib-0108] Richardson, K.M. , Griffin, P.C. , Lee, S.F. , Ross, P.A. , Endersby‐Harshman, N.M. , Schiffer, M. , and Hoffmann, A.A. (2019) A *Wolbachia* infection from *Drosophila* that causes cytoplasmic incompatibility despite low prevalence and densities in males. Heredity 122: 428–440. 30139962 10.1038/s41437-018-0133-7 PMC6460763

[emi15847-bib-0109] Roehrdanz, R.L. , and Wichmann, S.G.S. (2013) *Wolbachia* wsp gene clones detect the distribution of *Wolbachia* variants and wsp hypervariable regions among individuals of a multistrain infected population of *Diabrotica barberi* (Coleoptera: Chrysomelidae). Ann Entomol Soc Am 106: 329–338.

[emi15847-bib-0110] Roswell, M. , Dushoff, J. , and Winfree, R. (2021) A conceptual guide to measuring species diversity. Oikos 130: 321–338.

[emi15847-bib-0111] Rothman, J.A. , Cox‐Foster, D.L. , Andrikopoulos, C. , and McFrederick, Q.S. (2020) Diet breadth affects bacterial identity but not diversity in the pollen provisions of closely related Polylectic and Oligolectic bees. Insects 11: 645. 10.3390/insects11090645 PMC7564857 32962223

[emi15847-bib-0112] Salem, H. , Bauer, E. , Kirsch, R. , Berasategui, A. , Cripps, M. , Weiss, B. , *et al*. (2017) Drastic genome reduction in an herbivore's pectinolytic symbiont. Cell 171: 1520–1531. 29153832 10.1016/j.cell.2017.10.029

[emi15847-bib-0113] Salem, H. , Kirsch, R. , Pauchet, Y. , Berasategui, A. , Fukumori, K. , Moriyama, M. , *et al*. (2020) Symbiont digestive range reflects host plant breadth in herbivorous beetles. Curr Biol 30: 2875–2886. 32502409 10.1016/j.cub.2020.05.043

[emi15847-bib-0114] Santos‐Garcia, D. , Mestre‐Rincon, N. , Zchori‐Fein, E. , and Morin, S. (2020) Inside out: microbiota dynamics during host‐plant adaptation of whiteflies. ISME J 14: 847–856. 31896788 10.1038/s41396-019-0576-8 PMC7031279

[emi15847-bib-0115] Shannon, C.E. (1948) A mathematical theory of communication. Bell Syst Tech J 27: 379–423.

[emi15847-bib-0116] Shigenobu, S. , and Wilson, A.C. (2011) Genomic revelations of a mutualism: the pea aphid and its obligate bacterial symbiont. Cell Mol Life Sci 68: 1297–1309. 21390549 10.1007/s00018-011-0645-2 PMC3064905

[emi15847-bib-0117] Shukla, S.P. , and Beran, F. (2020) Gut microbiota degrades toxic isothiocyanates in a flea beetle pest. Mol Ecol 29: 4692–4705. 33006166 10.1111/mec.15657

[emi15847-bib-0118] Simpson, E.H. (1949) Measurement of diversity. Nature 163: 688–688.

[emi15847-bib-0119] Stammer, H.J. (1935) Studien an Symbiosen zwischen Käfern und Mikroorganismen. I. Die Symbiose der Donaciinen (Coleopt. Chrysomel.). Z Morphol Ökol Tiere 29: 585–608.

[emi15847-bib-0120] Stammer, H.J. (1936) Studien an Symbiosen zwischen Käfern und Mikroorganismen. II Die Symbiose des *Bromius obscurus* L. und der *Cassida* arten. (Coleopt. Chrysomel). Z Morphol Ökol Tiere 31: 682–697.

[emi15847-bib-0121] Takano, S. , Tuda, M. , Takasu, K. , Furuya, N. , Imamura, Y. , Kim, S. , *et al*. (2017) Unique clade of alphaproteobacterial endosymbionts induces complete cytoplasmic incompatibility in the coconut beetle. Proc Natl Acad Sci U S A 114: 6110–6115. 28533374 10.1073/pnas.1618094114 PMC5468645

[emi15847-bib-0122] Tayade, D.S. , Rawat, R.R. , and Chundurwar, R.D. (1975) First record of caecal diverticula in *Sagra femorata* Drury (Sagrinae: Coleoptera). Curr Sci 45: 812.

[emi15847-bib-0123] Ventura, C. , Briones‐Roblero, C.I. , Hernández, E. , Rivera‐Orduña, F.N. , and Zúñiga, G. (2018) Comparative analysis of the gut bacterial community of four *Anastrepha* fruit flies (Diptera: Tephritidae) based on pyrosequencing. Curr Microbiol 75: 966–976. 29520512 10.1007/s00284-018-1473-5

[emi15847-bib-0124] Wang, J. , Gao, Z. , Yang, M. , Xue, R. , Yan, H. , Fu, K. , *et al*. (2019) Geographically isolated Colorado potato beetle mediating distinct defense responses in potato is associated with the alteration of gut microbiota. J Pest Sci 93: 379–390.

[emi15847-bib-0125] Wang, S. , Wang, L. , Fan, X. , Yu, C. , Feng, L. , *et al*. (2020) An insight into diversity and functionalities of gut microbiota in insects. Curr Microbiol 77: 1976–1986. 32535651 10.1007/s00284-020-02084-2

[emi15847-bib-0126] Wei, J. , Segraves, K.A. , Li, W.Z. , Yang, X.K. , and Xue, H.J. (2020) Gut bacterial communities and their contribution to performance of specialist *Altica* flea beetles. Microb Ecol 80: 946–959. 32880699 10.1007/s00248-020-01590-x

[emi15847-bib-0127] Xu, L. , Sun, L. , Zhang, S. , Wang, S. , and Lu, M. (2019) High‐resolution profiling of gut bacterial communities in an invasive beetle using PacBio SMRT sequencing system. Insects 10: 248. 10.3390/insects10080248 PMC6722854 31416137

[emi15847-bib-0128] Yun, J.H. , Roh, S.W. , Whon, T.W. , Jung, M.J. , Kim, M.S. , Park, D.S. , *et al*. (2014) Insect gut bacterial diversity determined by environmental habitat, diet, developmental stage, and phylogeny of host. Appl Environ Microbiol 80: 5254–5264. 24928884 10.1128/AEM.01226-14 PMC4136111

[emi15847-bib-0129] Zhang, F. , Yang, C. , Zhang, X. , Zhu, H. , Zhao, D. , and Huang, Y. (2020a) Isolation of an anti‐entomopathogenic fungal protein secreted from *Pseudomonas aeruginosa* BGf‐2: an intestinal bacterium of *Blattella germanica* (L.). J Invertebr Pathol 107: 107371. 10.1016/j.jip.2020.107371 32268152

[emi15847-bib-0130] Zhang, J. , Ding, X. , Guan, R. , Zhu, C. , Xu, C. , Zhu, B. , *et al*. (2018) Evaluation of different 16S rRNA gene V regions for exploring bacterial diversity in a eutrophic freshwater lake. Sci Total Environ 618: 1254–1267. 29089134 10.1016/j.scitotenv.2017.09.228

[emi15847-bib-0131] Zhang, S. , Shu, J. , Xue, H. , Zhang, W. , Zhang, Y. , Liu, Y. , *et al*. (2020b) The gut microbiota in camellia weevils are influenced by plant secondary metabolites and contribute to saponin degradation. mSystems 5: e00692‐19. 32184361 10.1128/mSystems.00692-19 PMC7380582

[emi15847-bib-0132] Ziganshina, E.E. , Mohammed, W.S. , Shagimardanova, E.I. , Vankov, P.Y. , Gogoleva, N.E. , and Ziganshin, A.M. (2018) Fungal, bacterial, and archaeal diversity in the digestive tract of several beetle larvae (Coleoptera). BioMed Res Int 2018: 6765438. 29850548 10.1155/2018/6765438 PMC5926521

