# Supplementary figures and images for: Does diet breadth affect the complexity of the phytophagous insect microbiota? The case study of Chrysomelidae
Source: Environ Microbiol. 2021 Nov 30;24(8):3565–79. doi: 10.1111/1462-2920.15847 (PMC9543054; doi:10.1111/1462-2920.15847)

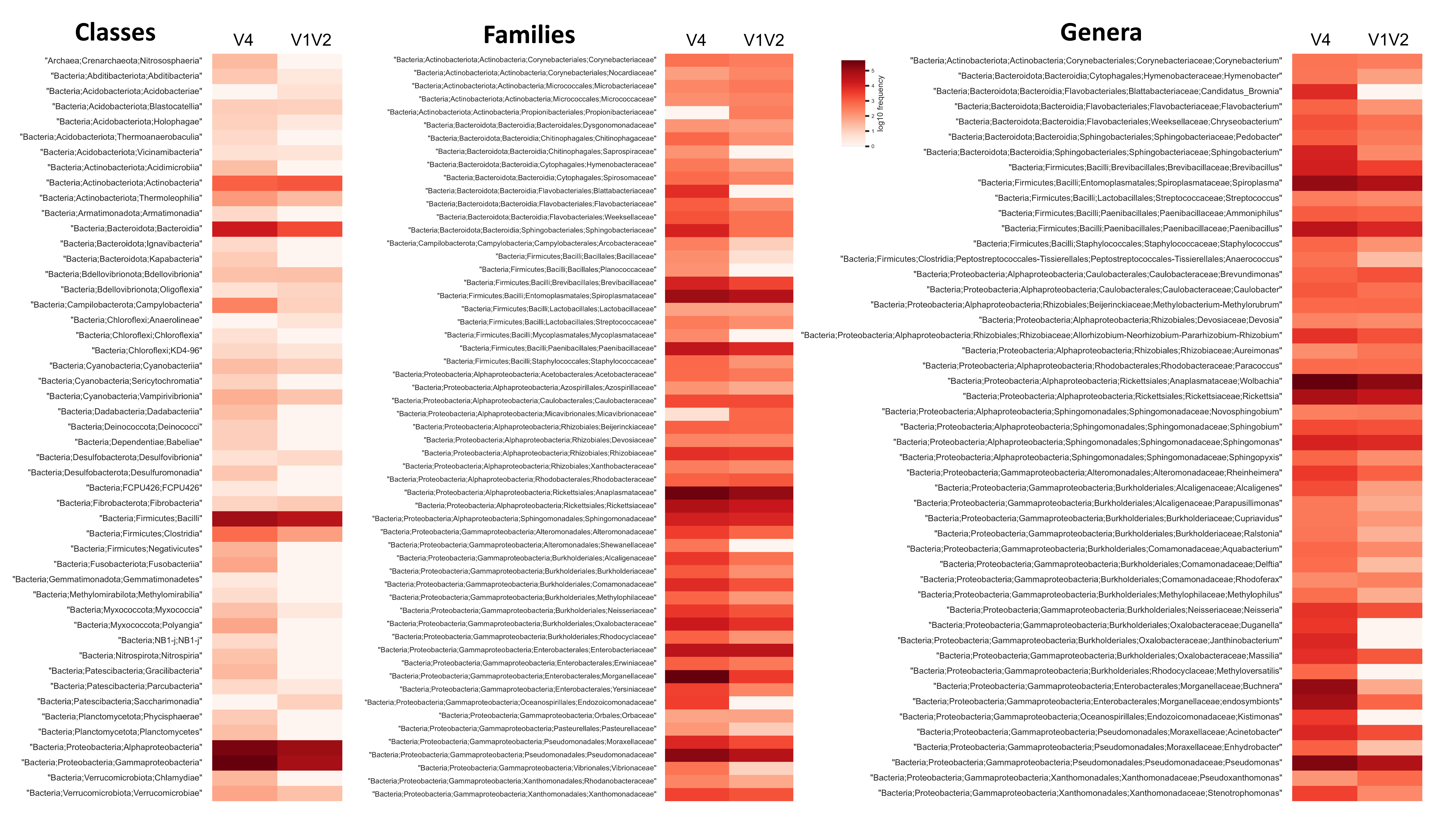

Supplement: Supplementary file 1 — Supplementary Fig. 1. Bacteria abundance in single marker datasets. Heatmap representing the abundance of bacterial taxa (classes, families, genera) present in the single marker datasets (V4 and V1‐V2). In the genera heatmap only the 50 most abundant genera are shown. Colour intensity is proportional to the normalized relative abundance of the bacterial taxa. [file EMI-24-3565-s003.tif]

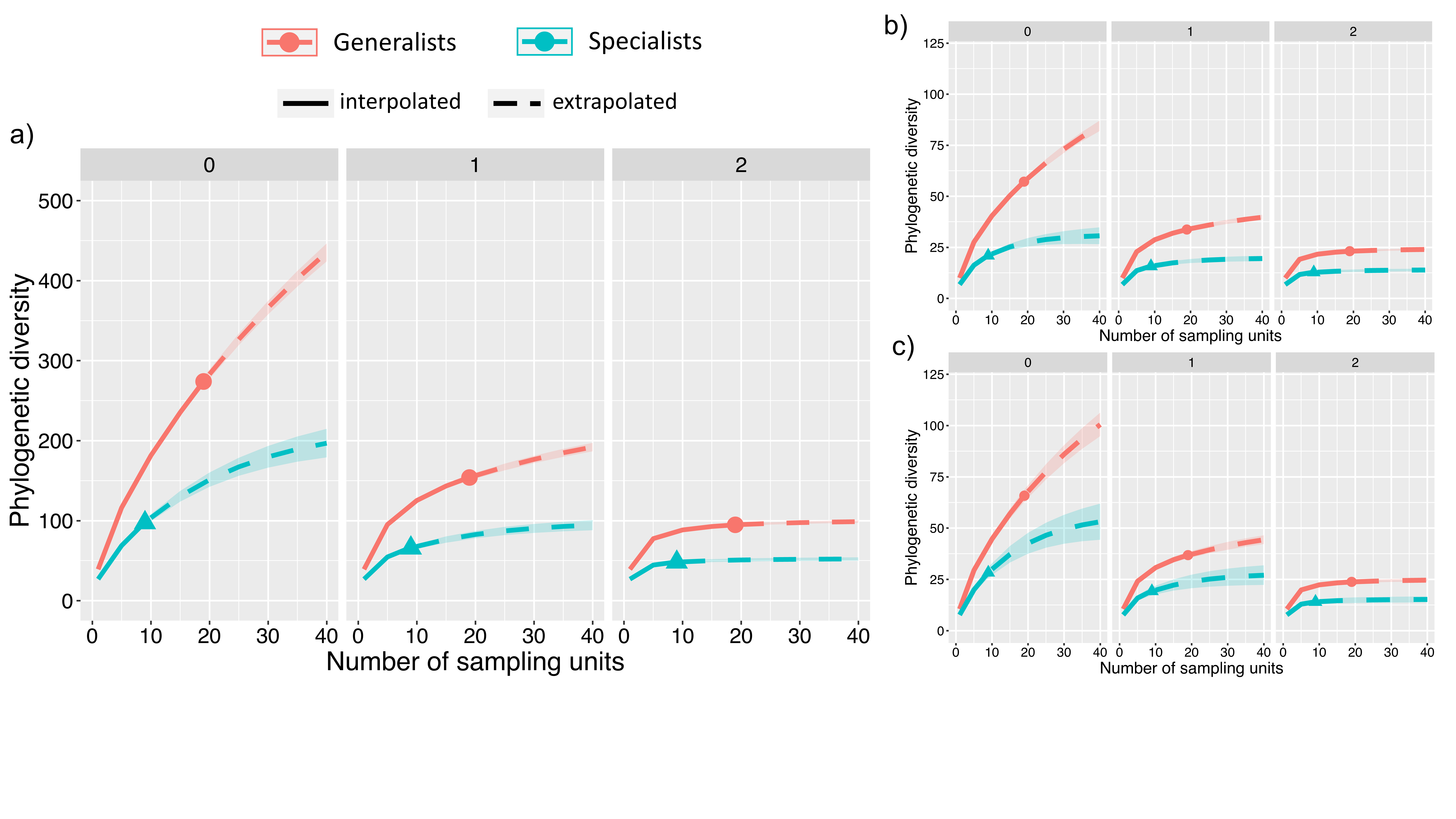

Supplement: Supplementary file 2 — Supplementary Fig. 2. Microbiota diversity estimates inferred on the total dataset (V1‐V2 and V4), V1‐V2 and V4 regions of the 16S rRNA. Sample‐based rarefaction/extrapolation curves of the Hill numbers estimated for three values of the order parameter (q = 0, q = 1, q = 2). The x‐axis represents increasing sampling and the y‐axis represents the Hill number estimates, 95% confidence interval is also reported. As reported in the legend, colours correspond to the trophic category (specialist or generalist) and line type to the methodological approach (interpolation or extrapolation). a) Global dataset (V1‐V2 and V4 regions of the16S rRNA). b) V1‐V2 region of the 16S rRNA. c) V4 region of the 16S rRNA. [file EMI-24-3565-s002.tif]

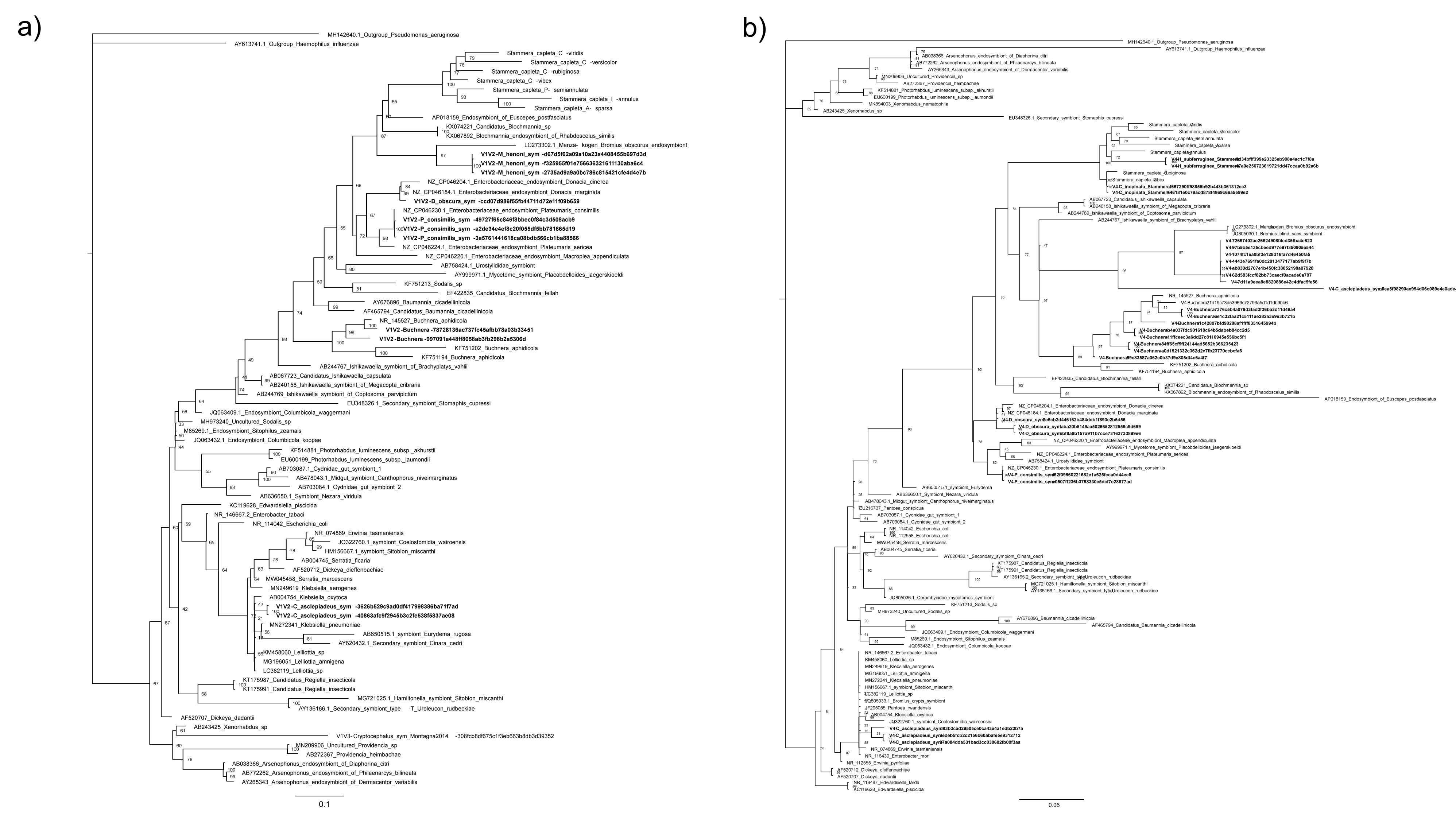

Supplement: Supplementary file 3 — Supplementary Fig. 3. Maximum likelihood phylogenetic trees. Sequences obtained from the NCBI database report the accession numbers while sequences produced in this study are highlighted in bold. a) Tree obtained from sequences of the V1‐V2 region of the 16S rRNA. b) Tree obtained from sequences of the V4 region of the 16S rRNA. [file EMI-24-3565-s001.tif]

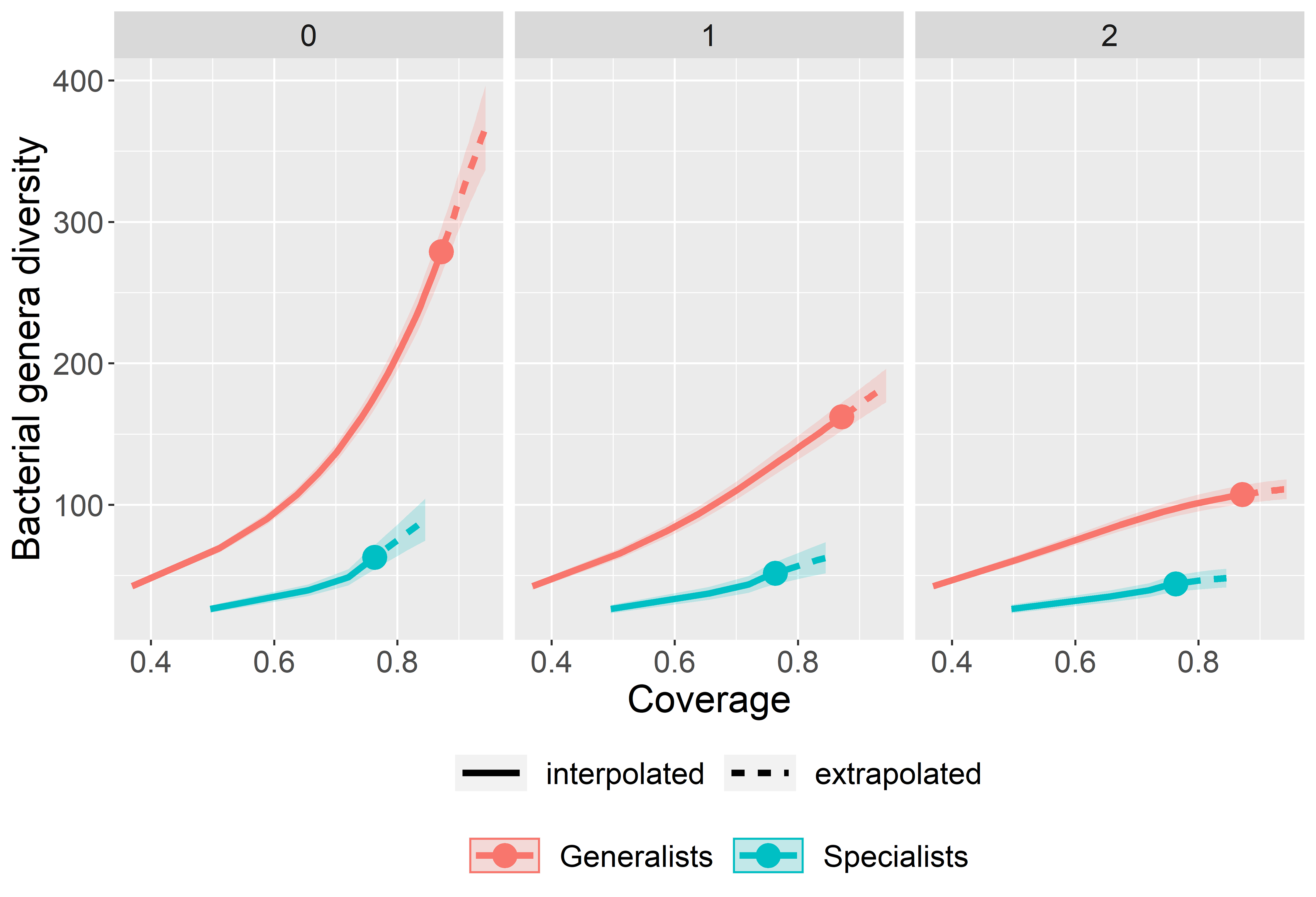

Supplement: Supplementary file 4 — Supplementary Fig. 4. Microbiota diversity of specialist and generalist Chrysomelidae defined using the plant taxonomic level of genus (specialists feed on plants all belonging to the same genus, generalists feed on plants belonging to different genera). Coverage based rarefaction/extrapolation curves of the Hill numbers estimated for three values of the order parameter (q = 0, q = 1, q = 2). The x‐axis represents the coverage (that estimates the completeness of the sampling) and the y‐axis represents the Hill number estimates, 95% confidence interval is also reported. As reported in the legend, colours correspond to the trophic category (specialist or generalist) and line type to the methodological approach (interpolation or extrapolation). [file EMI-24-3565-s005.tif]
