# Supplementary material for: Does diet breadth affect the complexity of the phytophagous insect microbiota? The case study of Chrysomelidae
Source: Environ Microbiol. 2021 Nov 30;24(8):3565–79. doi: 10.1111/1462-2920.15847 (PMC9543054; doi:10.1111/1462-2920.15847)
Supplement: Supplementary file 5 — Supplementary Table 1. Primary symbionts relative abundance. Supplementary Table 2. Blast search results on NCBI nt database. Supplementary Table 3. Accession numbers of COI sequences. [file EMI-24-3565-s004.docx]

Supplementary table 1. Primary symbionts relative abundance.

|  | *Wolbachia* | *Rickettsia* | *Spiroplasma* | *Buchnera* | *Ca.* Stammera capleta | Donacinae endosymbiont |
| --- | --- | --- | --- | --- | --- | --- |
| *Cassida inopinata* | <0.1% |  |  |  | 74.7% |  |
| *Luperus longicornis* | 94.8% | 3.3% | <0.1% | <0.1% |  |  |
| *Chrysolina fastuosa* | 0.15% | 9.2% | <0.1% | <0.1% |  |  |
| *Smaragdina afffinis* | 5.8% | 92.1% |  | <0.1% |  |  |
| *Cryptocephalus loreyi* | 0.65% |  |  |  |  |  |
| *Cryptocephalus transcaucasicus* | 0.11% |  | <0.1% | <0.1% |  |  |
| *Crepidodera fulvicornis* | 0.34% | <0.1% |  | <0.1% |  |  |
| *Labidostomis longimana* | <0.1% | 18.1% | <0.1% | <0.1% |  |  |
| *Altica oleracea* | 0.75% | 0.35% | <0.1% | <0.1% |  |  |
| *Chaetocnema hortensis* | 70.6% |  | <0.1% | <0.1% |  |  |
| *Pachybrachis exclusus* | 96% |  | <0.1% |  |  |  |
| *Cryptocephalus fulvus* | 0.11% |  | <0.1% |  |  |  |
| *Clytra quadripunctata* | <0.1% | 43.8% | <0.1% | <0.1% |  |  |
| *Lilioceris merdigera* | <0.1% | <0.1% | 0.65% |  |  |  |
| *Hispa atra* | 1.0% | 31% |  |  |  |  |
| *Dicladispa testacea* | 63.5% |  |  | <0.1% |  |  |
| *Donacia obscura* | 41% |  |  | <0.1% |  | 55.3% |
| *Chrysomela saliceti* |  |  | <0.1% | <0.1% |  |  |
| *Exosoma thoracicum* | 98.9% |  |  | <0.1% |  |  |
| *Zeugophora flavicollis* | 95.2% |  | <0.1% | <0.1% |  |  |
| *Timarcha tenebricosa* |  |  |  |  |  |  |
| *Orsodacne humeralis* | 0.18% |  | 0.74% | 22.1% |  |  |
| *Plateumaris consimilis* | 5.7% |  |  | <0.1% |  | 93.8% |
| *Calligrapha* sp. | <0.1% |  |  |  |  |  |
| *Macrocoma henoni* | <0.1% |  | <0.1% |  |  |  |
| *Prasocuris phellandrii* | 82% |  |  | <0.1% |  |  |
| *Crioceris paracenthesis* | <0.1% |  | 97.63% | 0.12% |  |  |
| *Chrysochus asclepiadeus* | <0.1% |  | <0.1% | <0.1% |  |  |
| *Orsodacne cerasi* | <0.1% |  |  |  |  |  |
| *Hypocassida subferruginea* | <0.1% |  | <0.1% | <0.1% | 93.1% |  |

Supplementary table 2. Blast search results on NCBI nt database.

| Species | 16S region | ASV ID | BLAST top hits | Coverage | Identity | Accession numbers |
| --- | --- | --- | --- | --- | --- | --- |
| *D. marginata* | V1V2 | ccd07d986f55fb44711d72e11f09b659 | Enterobacteriaceae endosymbiont of *Donacia marginata* isolate DmarSym chromosome | 100% | 97.72% | CP046184.1 |
| *D. marginata* | V4 | 2e6cb2d446162b484ddb1f893e2b5d56 | Enterobacteriaceae endosymbiont of *Donacia marginata* isolate DmarSym chromosome | 100% | 99.24% | CP046184.1 |
| *D. marginata* | V4 | faba20b5149aa5026652812559c9d699 | Enterobacteriaceae endosymbiont of *Donacia marginata* isolate DmarSym chromosome | 100% | 98.09% | CP046184.1 |
| *D. marginata* | V4 | bbf8a9b157a911b7cce73163733899e6 | Enterobacteriaceae endosymbiont of *Donacia marginata* isolate DmarSym chromosome | 100% | 98.47% | CP046184.1 |
| *P. consimilis* | V1V2 | a2de34e4ef8c20f055df5bb781665d19 | Enterobacteriaceae endosymbiont of *Plateumaris consimilis* isolate PconSym chromosome | 100% | 99.67% | CP046230.1 |
| *P. consimilis* | V1V2 | 49727f65c846f8bbec0f84c3d508acb9 | Enterobacteriaceae endosymbiont of *Plateumaris consimilis* isolate PconSym chromosome | 100% | 99.35% | CP046230.1 |
| *P. consimilis* | V1V2 | 3a5761441618ca08bdb566cb1ba88566 | Enterobacteriaceae endosymbiont of *Plateumaris consimilis* isolate PconSym chromosome | 100% | 99.67% | CP046230.1 |
| *P. consimilis* | V4 | f62f09560221682e1a625fcca0d44ee8 | Enterobacteriaceae endosymbiont of *Plateumaris consimilis* isolate PconSym chromosome | 100% | 99.62% | CP046230.1 |
| *P. consimilis* | V4 | e0507ff236b3798330e5dcf7e28877ad | Enterobacteriaceae endosymbiont of *Plateumaris consimilis* isolate PconSym chromosome | 100% | 100.00% | CP046230.1 |
| *C. inopinata* | V4 | ef667290ff98855b92b443b361312ec3 | *Candidatus* Stammera capleta isolate NZ1215 chromosome, complete genome | 100% | 98.47% | CP024013.1 |
| *C. inopinata* | V4 | 646181e0c79acd878f4869c66a5599e2 | *Candidatus* Stammera capleta isolate NZ1215 chromosome, complete genome | 100% | 98.85% | CP024013.1 |
| *H. subferruginea* | V4 | 0d34bfff399e23325eb998a4ac1c7f8a | *Candidatus* Stammera capleta isolate NZ1215 chromosome, complete genome | 100% | 95.42% | CP024013.1 |
| *H. subferruginea* | V4 | 47a0e256723619721dd47ccaa0b92a6b | *Candidatus* Stammera capleta isolate NZ1215 chromosome, complete genome | 100% | 95.80% | CP024013.1 |
| *C. asclepiadeus* | V1V2 | 3626b529c9ad0df417998386ba71f7ad | *Lelliottia amnigena* strain NCTC12124 genome assembly, chromosome: 1 | 100% | 98.37% | LR134135.1 |
| *C. asclepiadeus* | V1V2 | 40863afc9f2945b3c2fe538f5837ae08 | *Lelliottia amnigena* strain NCTC12124 genome assembly, chromosome: 1 | 100% | 98.70% | LR134135.1 |
| *C. asclepiadeus* | V4 | 7edeb5fcb2c2156b60abafe5e9312712 | *Klebsiella aerogenes* strain K64 16S ribosomal RNA gene, partial sequence | 100% | 98.47% | MN860163.1 |
| *C. asclepiadeus* | V4 | 97a084dda531bad3cc838682fb00f3aa | *Klebsiella aerogenes* strain K64 16S ribosomal RNA gene, partial sequence | 100% | 98.85% | MN860163.1 |
|  |  |  | *Klebsiella aerogenes* strain NCTC9735 genome assembly, chromosome: 1 | 100% | 98.85% | LR134475.1 |
| *C. asclepiadeus* | V4 | d83b3cad29505ce0ca43e4a1edb23b7a | *Klebsiella variicola* strain EM09 16S ribosomal RNA gene, partial sequence | 100% | 98.09% | MT279983.1 |
|  |  |  | *Klebsiella pneumoniae* strain KlPn 3 16S ribosomal RNA gene, partial sequence | 100% | 98.09% | MT255043.1 |
| *C. asclepiadeus* | V4 | c5ea5f98290ae954d06c089e4e0ade41 | Gamma proteobacterium Manza-kogen gene for 16S ribosomal RNA, partial sequence | 99% | 87.36% | LC273302.1 |
|  |  |  | Uncultured bacterium clone *Bromius*_blind_sacs_symbiont 16S ribosomal RNA gene, partial sequence | 99% | 87.36% | JQ805030.1 |
| *M. henoni* | V1V2 | d67d5f62a09a10a23a4408455b697d3d | Endosymbiont of *Euscepes postfasciatus* DNA, complete genome, isolate: NAREPO1 | 100% | 78.50% | AP018159.1 |
|  |  |  | *Blochmannia* endosymbiont of *Rhabdoscelus similis* clone NAN-4 16S ribosomal RNA gene, partial sequence | 100% | 78.30% | KX067892.1 |
| *M. henoni* | V1V2 | f325955f01e756636321611130aba6c4 | Endosymbiont of *Euscepes postfasciatus* DNA, complete genome, isolate: NAREPO1 | 100% | 79.15% | AP018159.1 |
|  |  |  | *Blochmannia* endosymbiont of Rhabdoscelus similis clone NAN-4 16S ribosomal RNA gene, partial sequence | 100% | 78.93% | KX067892.1 |
| *M. henoni* | V1V2 | 2735ad9a9a0bc786c815421cfe4d4e7b | Endosymbiont of *Euscepes postfasciatus* DNA, complete genome, isolate: NAREPO1 | 100% | 78.83% | AP018159.1 |
|  |  |  | *Blochmannia* endosymbiont of *Rhabdoscelus similis* clone NAN-4 16S ribosomal RNA gene, partial sequence | 100% | 78.62% | KX067892.1 |
| *M. henoni* | V4 | ab830d2707e1b450fc38852198a07928 | Gamma proteobacterium Manza-kogen gene for 16S ribosomal RNA, partial sequence | 100% | 93.89% | LC273302.1 |
|  |  |  | Uncultured bacterium clone *Bromius*_blind_sacs_symbiont 16S ribosomal RNA gene, partial sequence | 100% | 93.89% | JQ805030.1 |
| *M. henoni* | V4 | 62d583fccf82bb73caecf0acade0a797 | Gamma proteobacterium Manza-kogen gene for 16S ribosomal RNA, partial sequence | 100% | 93.51% | LC273302.1 |
|  |  |  | Uncultured bacterium clone *Bromius*_blind_sacs_symbiont 16S ribosomal RNA gene, partial sequence | 100% | 93.51% | JQ805030.1 |

Supplementary table 3. Accession numbers of COI sequences.

| Species | Accession Number |
| --- | --- |
| *Altica oleracea* | JF890683 |
| *Cassida inopinata* | JF890687 |
| *Chaetocnema hortensis* | JF890767 |
| *Chrysochus asclepiadeus* | JF890698 |
| *Chrysolina fastuosa* | JF890727 |
| *Chrysomela saliceti* | MH322815 |
| *Clytra quadripunctata* | JF890821 |
| *Crepidodera fulvicornis* | JF890763 |
| *Crioceris paracenthesis* | MH322856 |
| *Cryptocephalus fulvus* | MH322918 |
| *Cryptocephalus loreyi* | JF890726 |
| *Cryptocephalus transcaucasicus* | LS973870 |
| *Dicladispa testacea* | MH323090 |
| *Donacia obscura* | MH323097 |
| *Exosoma thoracicum* | MH323108 |
| *Hispa atra* | MH323146 |
| *Hypocassida subferruginea* | JF890707 |
| *Labidostomis longimana* | MH323150 |
| *Lilioceris merdigera* | JF890824 |
| *Luperus longicornis* | JF890701 |
| *Macrocoma henoni* | MH323229 |
| *Orsodacne cerasi* | JF890673 |
| *Orsodacne humeralis* | MH323283 |
| *Pachybrachis exclusus* | JF890775 |
| *Plateumaris consimilis* | KM450130 |
| *Prasocuris phellandrii* | JF890801 |
| *Smaragdina afffinis* | MH323362 |
| *Timarcha tenebricosa* | MH323399 |
| *Zeugophora flavicollis* | MH323403 |
